# Advances and Perspectives in Graphene‐Based Quantum Dots Enabled Neuromorphic Devices

**DOI:** 10.1002/advs.202600042

**Published:** 2026-03-20

**Authors:** Yulin Zhen, Wei Zeng, Zherui Zhao, JiYu Zhao, Ziyan Huang, Zhuo Chen, Kui Zhou, Guanglong Ding, Su‐Ting Han, Ye Zhou

**Affiliations:** ^1^ Institute For Advanced Study Shenzhen University Shenzhen P. R. China; ^2^ State Key Laboratory of Fine Chemicals Frontiers Science Center for Smart Materials Dalian University of Technology Dalian P. R. China; ^3^ State Key Laboratory of Radio Frequency Heterogeneous Integration Shenzhen University Shenzhen P. R. China; ^4^ Department of Applied Biology and Chemical Technology The Hong Kong Polytechnic University Kowloon Hong Kong P. R. China

**Keywords:** artificial synapses, brain‐inspired computing, graphene‐based quantum dots, neuromorphic devices, non‐volatile memories

## Abstract

With the rapid rise in demand for artificial intelligence and brain‐inspired computing, traditional von Neumann architecture is gradually approaching physical limitations in terms of computing power density, energy efficiency, and real‐time performance. Graphene quantum dots (GQDs) and graphene oxide quantum dots (GOQDs), as zero‐dimensional carbon‐based materials with quantum confinement effects and tunable band structures, have shown significant potential in building next‐generation ultra‐low‐power, large‐scale integrated neuromorphic devices. This review systematically summarizes the main preparation strategies of graphene‐based QDs and their structural regulation and functionalization methods. It focuses on the core functions of graphene‐based QDs in synaptic working mechanisms such as charge capture, ion migration, and optoelectronic cooperation, as well as their latest progress in non‐volatile memories, electrical and optoelectronic artificial synapses, and neuromorphic systems. Finally, this review article summarizes the current key challenges from the perspectives of material controllability, mechanism interpretability, device structural engineering, and system‐level heterogeneous integration, and proposes future research directions to provide reference for the development of next‐generation high‐efficiency, scalable brain‐inspired computing hardware.

## Graphical abstract

Graphene‐based QDs are zero‐dimensional carbon nanomaterials with pronounced quantum confinement and tunable electronic structures. Herein, we summarize their synthesis strategies and functionalization methods, and highlight their functional roles and operating mechanisms in devices, as well as recent advances in neuromorphic electronics. We anticipate that this review may promote the development of graphene‐based QDs for future high‐efficiency neuromorphic computing systems.

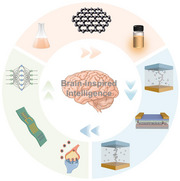

**Abbreviations**
BP: Black phosphorus
CEA: Chicken egg albumin
CF: Carbon fibers
CFs: Conductive filaments
EPSC: Excitatory postsynaptic current
GQDs: Graphene quantum dots
HOMO: Highest occupied molecular orbital
HRS: High‐resistance state
I_DS_
: Source–drain current
LRS: Low‐resistance state
LTD: Long‐term depression
LTM: Long‐term memory
LTP: Long‐term potentiation
PMMA: Poly(methyl methacrylate)
PPF: Paired‐pulse facilitation
PVP: Polyvinylpyrrolidone
RRAM: Resistive Random‐Access Memory
STDP: Spike‐timing‐dependent plasticity
STM: Short‐term memory
STP: Short‐term plasticity
UV: Ultraviolet
V_GS_
: Gate voltage
V_TH_
: Threshold voltage

## 1 Introduction

Driven by the rapid development of artificial intelligence in recent years, particularly in the field of deep learning, contemporary society is undergoing an exponential growth phase in the generation, transmission, and processing of global data [1, 2]. Against this backdrop, real‐world application scenarios have imposed higher demands on large‐capacity and high‐bandwidth information transmission mechanisms, as well as on efficient information‐processing technologies. These developments have fundamentally reshaped our understanding of, and requirements for, storage capabilities and computational performance [3, 4]. The traditional von Neumann architecture, with its core design of physical separation between memory and processor, is facing significant challenges. The gradual failure of Moore's Law, along with multiple bottlenecks such as the “memory wall” and the “power wall,” are restricting its performance and adaptability in advanced computing scenarios [5, 6]. Therefore, in response to the urgent demand for low‐energy, highly parallel, and efficient information processing, the post‐Moore's Law era necessitates the development of brain‐inspired computing paradigms based on new principles, new processes, and new material electronic devices [7, 8, 9].

In this trend, the asynchronous, multimodal, and highly fault‐tolerant information processing mechanism of the human brain's nervous system provides important insights for overcoming traditional computing bottlenecks [10, 11]. The human brain, an extraordinarily complex parallel computing system, operates on merely 20 watts of power and utilizes neuronal action potentials of approximately 100 mV. Despite this remarkably low energy consumption, it can easily accomplish sophisticated cognitive tasks that current supercomputers, which require megawatt‐level power, are only able to perform with great difficulty [12]. Researchers have introduced concepts such as neuromorphic computing and artificial neural networks to emulate the structure and function of the human brain. By simulating the structure and plasticity mechanisms of neurons and synapses in the brain, they have designed novel hardware and software algorithms [13, 14, 15]. Early research primarily used CMOS analog circuits to simulate the functions of biological synapses, attempting to replicate the information processing mechanisms of the brain on silicon‐based platforms. Although the CMOS process nodes have continuously shrunk, significantly increasing integration scale, the approach of relying on traditional transistor stacking to achieve biological synapse functions has resulted in a high transistor‐to‐synapse ratio. For example, in TrueNorth, about 21 transistors are required for each synapse, and this ratio has not significantly decreased even with more advanced processes [16, 17]. This design paradigm, which sacrifices hardware scale for functional implementation, has gradually improved in terms of scale but is fundamentally limited by high energy consumption, expensive hardware costs, and low area efficiency. These limitations make it difficult for traditional silicon‐based transistors to achieve high density integration in artificial neural networks. On the other hand, neuromorphic devices, by mimicking the structure and functional principles of biological neural systems (especially the human brain), can directly simulate the behavior of neurons and synapses in the brain from a physical level, relying on their unique mechanisms. In contrast, the highly parallel and in‐memory computing integration features of neuromorphic devices provide a new pathway for constructing neuromorphic systems with extremely high computing ability and memory‐computing integrated architectures [18, 19, 20, 21, 22]. However, current artificial neuromorphic devices based on traditional materials (such as metal oxides and chalcogenide compounds) face significant challenges when scaled down to the nanoscale, particularly with respect to the thickness of the functional layers. The uncertainty in interface effects and the distribution of defect states can significantly affect the uniformity and stability of device performance, leading to a series of issues, including a decrease in the reliability of neuromorphic functions and a sharp increase in power density [23, 24]. Therefore, to address the interface effects and performance fluctuations faced by existing neuromorphic devices at the nanoscale, the development of novel low‐dimensional functional materials with precise energy level regulation, excellent interface compatibility, and good size uniformity has become an urgent research direction in this field [25, 26, 27].

In recent years, quantum dots (QDs), as a representative class of zero‐dimensional materials, have attracted significant attention in the fields of non‐volatile memory technology, optoelectronic devices, and neuromorphic computing, owing to their unique optical and electrical properties [28, 29, 30, 31, 32]. Among them, carbon‐based QDs, due to their excellent biocompatibility, good stability, and ease of functional modification, have shown great potential in constructing low‐power biomimetic synaptic devices. Graphene‐based QDs are a class of zero‐dimensional carbon nanomaterials derived from graphene, primarily encompassing GQDs and GOQDs. GQDs, as a representative subclass, consist of single‐layer or few‐layer sp^2^‐hybridized carbon sheets confined in all three spatial dimensions at the nanoscale (typically with lateral sizes below 20 nm). Distinct from conventional quasi‐spherical or amorphous carbon dots (such as carbon quantum dots (CQDs) and polymer dots), GQDs retain the ordered sp^2^‐conjugated crystalline structure of graphene [33, 34]. Their optoelectronic properties are primarily governed by quantum confinement and edge states rather than surface defects [35]. In addition, GQDs exhibit a larger specific surface area and higher, more stable quantum yields (QY), whereas conventional CQDs typically show QY below 20% [36, 37, 38, 39]. These structural features endow GQDs with a more well‐defined and tunable band structure, superior carrier mobility, and enhanced stability of optoelectronic responses [40, 41]. Compared to traditional semiconductor quantum dots (such as CdSe and PbS), GQDs, as an emerging class of quantum materials, offer advantages including high biocompatibility without heavy metals, strong stability from modifiable functional groups, and flexible conductivity characteristics that can be controlled in multiple dimensions [42]. In addition, GQDs have low preparation costs and good compatibility with silicon‐based processes, making them better suited for use in devices that demand low toxicity, long operational lifespans, and high‐precision synaptic simulation, as well as large‐scale integration [43, 44]. Furthermore, studies have shown that using a multi‐quantum dot structure can significantly reduce the Coulomb blockade region, thereby enhancing the current intensity and operating speed of single electron transistor [45]. Based on these novel properties, GQDs have attracted widespread attention in the academic community for their application in neuromorphic computing, providing a highly promising material system and innovative structural strategies for the development of high‐performance, energy‐efficient brain‐inspired computing devices.

**FIGURE 1:**
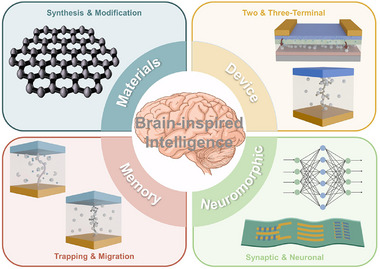
Overall diagram of neuromorphic device based on graphene‐based QDs.

This review comprehensively reviews and summarizes the recent research progress on graphene‐based QDs neuromorphic devices (Figure 1). First, we introduce the basic preparation methods of graphene‐based QDs and their key modification strategies. Then, we focus on discussing the main mechanisms of graphene‐based QDs in neuromorphic devices, such as charge capture, ion migration, and optoelectronic cooperation. Based on this, we systematically categorize various types of neuromorphic devices with graphene‐based QDs, and summarize their representative device performances and application examples. Finally, we discuss the key challenges currently faced from multiple dimensions, including materials, mechanisms, device structure, and system integration, and propose future development directions.

## 2 Preparation Methods of GQDs and GOQDs

**FIGURE 2:**
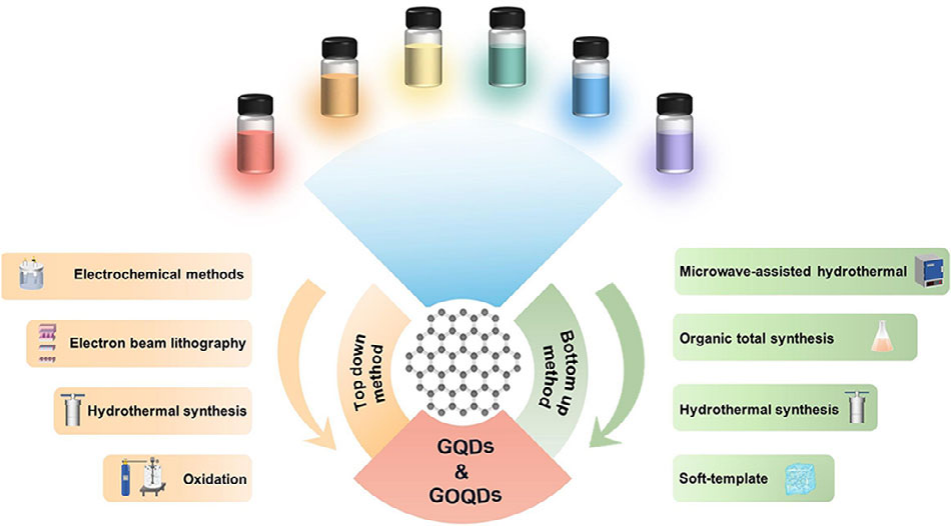
Synthesis methods of GQDs and GOQDs: top‐down and bottom‐up methods.

The size uniformity, doping type, defect density, and surface functional group characteristics of graphene‐based QDs are key structural parameters that influence the performance of neuromorphic devices. These parameters directly determine the response speed, cycle stability, and scalability of neuromorphic devices. The regulation of these key parameters largely depends on the choice of preparation method and process optimization. According to current research reports, GQDs and GOQDs are mainly prepared using two mainstream strategies: “top‐down” and “bottom‐up” approaches (Figure 2). Among these approaches, top‐down methods are the most widely employed for the preparation of GOQDs and oxidized GQDs, whereas bottom‐up strategies are more commonly adopted for the synthesis of high‐purity GQDs [46, 47, 48].

### 2.1 Top‐Down Method

The top‐down approach utilizes large‐scale carbon sources, such as carbon nanotubes, graphene oxide, and graphite, as raw materials. These materials are subsequently reduced to smaller structures through physical, chemical, or electrochemical exfoliation techniques, which disrupt the π‐π stacking between layers and the sp^2^ carbon bonds within the layers, gradually breaking the bulk carbon material into nanoscale fragments. Wu et al. successfully prepared GQDs using the top‐down strategy [49]. This method, while retaining the excellent intrinsic properties of the parent material, introduces the unique advantages of quantum confinement and edge effects. It not only demonstrates the feasibility of controllably converting three‐dimensional bulk materials into zero‐dimensional quantum structures through physical and chemical cutting, but also provides an important paradigm for pioneering research on other low‐dimensional materials.

The mainstream top‐down preparation methods for GQDs and GOQDs include liquid‐phase exfoliation methods assisted by oxidation, hydrothermal, electrochemical methods, or microwave‐ultrasonication, as well as electron beam lithography [50, 51]. The liquid‐phase exfoliation method mainly involves the use of strong oxidizers to intercalate graphite, followed by thermal expansion or chemical expansion methods to achieve interlayer separation of graphite. In recent years, the technology for preparing GQDs and GOQDs using this method has been continuously optimized. Wang et al. employed citric acid as a precursor and synthesized GOQDs with an average lateral size of approximately 3.8 nm via a facile hydrolysis route. The resulting GOQDs are enriched with polar oxygen‐containing functional groups, such as epoxy, hydroxyl, and carboxyl moieties, endowing them with excellent hydrophilicity and a negatively charged surface [52]. Juárez et al. reported a green synthesis of GOQDs using orange peel as the carbon source through a carbonization–ultrasonic exfoliation process. The obtained GOQDs exhibit an average size of ∼3 nm, display characteristic absorption features in UV–vis spectra, and show blue photoluminescence emission (≈2.8 eV) under 350 nm excitation, indicating favorable photoluminescent properties [53]. Chatterjee et al. prepared GOQDs from graphene oxide nanosheets via a nitric‐acid‐assisted oxidative cutting strategy under relatively well‐controlled conditions. The as‐synthesized GOQDs possess good water solubility; their aqueous solutions emit in the green/yellow region under acidic or near‐neutral conditions, while a strong orange‐red emission emerges at pH 12.8 with a relative photoluminescence quantum yield of approximately 30%. Moreover, these GOQDs exhibit excellent photostability and white‐light emission behavior when embedded in a poly(vinyl alcohol) (PVA) matrix [54]. Wang et al. used graphite as the raw material and prepared white fluorescent‐emitting GQDs (WGQDs) through a two‐step microwave‐assisted hydrothermal liquid‐phase exfoliation method. They subsequently fabricated a white‐light‐emitting diode (LED) using these WGQDs, achieving a record‐high external quantum efficiency of 0.2% for GQD at the time [55]. Zhang et al. combined liquid‐phase exfoliated GQDs with silk fibroin to construct a three‐state memristor. Through the interface barrier modulation of GQDs, they successfully achieved key synaptic functions such as paired‐pulse facilitation (PPF) and long‐term potentiation (LTP). The device demonstrated a low power consumption of 0.25 µW and achieved a recognition accuracy of 98.38% in the MNIST task [56]. Since the preparation of GQDs and GOQDs based on the liquid‐phase exfoliation method is carried out in a liquid environment, the physical and chemical properties of GQDs and GOQDs can be precisely controlled by adjusting the process environment and preparation parameters. However, this method may lead to solvent residues (such as NMP), which can affect the device's performance stability. Additionally, conditions such as humidity can influence the reliability of the device through edge functional groups. Based on previous research on carbon dots (CDs), Geim et al. successfully used electron beam lithography to prepare GQDs for the first time in 2008 [51]. The high‐resolution advantage of electron beam lithography enables precise control over the size and spatial distribution of GQDs, allowing for the arbitrary regulation of their diameter. This is crucial for the fabrication of neuromorphic devices with uniform performance. However, the reliance on expensive equipment for electron beam lithography limits its widespread application in the preparation of GQDs. Moreover, electron scattering may introduce defects, which can affect the quantum confinement effects of GQDs.

The top‐down method offers a wide range of raw materials and is cost‐effective, making it suitable for large‐scale production. In processes such as chemical oxidation, rich oxygen‐containing functional groups are introduced to the edges and surfaces of GQDs, enhancing their hydrophilicity and providing active sites for subsequent functional modifications. However, this method also has drawbacks, including a high number of defects and the issue of uneven product size and morphology.

### 2.2 Bottom‐Up Method

In recent years, significant progress has been made in the precise and controlled preparation of GQDs using the bottom‐up method. This approach uses small molecular precursors (such as citric acid, glucose, benzene derivatives, etc.) as raw materials, and synthesizes GQDs with specific structures through chemical reactions like pyrolysis and carbonization at the molecular level. The preparation strategies for this method include organic total synthesis, hydrothermal synthesis, microwave‐assisted hydrothermal synthesis, and soft‐template methods, among others [57, 58, 59, 60, 61, 62, 63].

Among these, organic total synthesis represents one of the most mainstream preparation strategies. This approach typically employs polycyclic aromatic hydrocarbons, polyphenyl dendritic molecules, or other designable small molecules as precursors, and achieves the controllable extension of π‐conjugated frameworks through stepwise skeleton growth combined with a series of rigorous organic reactions (e.g., coupling, cyclization, and oxidative condensation) [64, 65]. As a result, GQDs with pre‐designed size, edge structures, and functional groups can be obtained. Compared with other bottom‐up methods, this strategy is particularly crucial for realizing atomically precise synthesis of GQDs, elucidating structure–property relationships, and thereby enabling targeted tuning of neuromorphic device performance. In addition, organic total synthesis allows precise modulation of band structures, photoluminescence characteristics, and electrochemical behaviors by introducing specific functional groups and heteroatoms either during the stepwise framework construction or via post‐synthetic modification. Notably, Li et al., based on the Scholl oxidative condensation reaction combined with a new solubilization strategy, successfully prepared graphene quantum dots containing 168, 132, and 170 conjugated carbon atoms, representing the largest stable colloidal GQDs reported at that time [66]. Building on this work, researchers further utilized precise organic synthetic techniques to fabricate a series of nitrogen‐doped colloidal GQDs with controllable sizes and structures, laying an important foundation for the precise synthesis and mechanistic investigation of GQDs [67]. Despite its significant advantages in structural controllability, this strategy generally relies on multistep organic reactions, leading to relatively complex synthetic procedures and low overall yields, which still impose limitations on large‐scale production.

Zaidi et al. successfully synthesized GQDs using date palm leaves as a green precursor through an environmentally friendly hydrothermal method, providing a new pathway for the resource utilization of agricultural waste and the sustainable preparation of nanomaterials [68]. Tran et al. proposed an efficient microwave‐assisted hydrothermal synthesis strategy, achieving the rapid transformation of graphene oxide into monodispersed GQDs (2‐8 nm) within ten minutes. The products exhibited distinct blue fluorescence emission characteristics, significantly improving the preparation efficiency and process feasibility of GQDs [69]. Lau et al. successfully prepared GQDs with highly uniform sizes (<5 nm ± 0.55 nm) using the soft‐template method. They systematically revealed the size‐dependent photoluminescence quantum yield and carrier relaxation mechanisms, providing key experimental evidence for the precise analysis of the band structure of GQDs [57]. Among these approaches, the hydrothermal method is operationally straightforward but typically requires prolonged reaction times; the microwave‐assisted approach markedly shortens the reaction duration, yet offers limited precision in controlling size and morphology; meanwhile, the soft‐template method enables relatively precise size regulation, albeit with increased procedural complexity.

Compared to the top‐down method, the bottom‐up synthesis strategy allows for precise control over the size, morphology, edge structure, and surface functional groups of graphene‐based QDs by selecting specific precursors, thus enabling the production of GQDs with fewer defects, higher crystallinity, and excellent optoelectronic properties. This method has significant advantages in improving material consistency and reducing device electrical noise, which is beneficial for achieving high‐performance and high‐stability neuromorphic devices. However, the bottom‐up method also faces challenges such as poor solubility, complex synthesis, high costs, and difficulty in mass production. Therefore, in practical applications, the choice of the graphene‐based QDs preparation strategy must consider multiple factors. If performance consistency, precise conductivity control, and high stability (such as for high‐precision neuromorphic devices) are prioritized, the bottom‐up method with precise structural control is recommended. If cost and scalability (such as for disposable sensors) are more important, the simpler top‐down method can be selected.

## 3 Modification of GQDs

The modification of GQDs, by regulating their structure, surface functional groups, and electronic states, not only enhances the overall conjugation of the structure but also alters the photoluminescent properties, band structure, and chemical reactivity of GQDs. This can meaningfully improve their optical, chemical, and electrical properties, as well as their stability and dispersibility, enabling them to play a key role in high‐performance applications. Currently, the main modification methods for GQDs can be classified into three categories: surface functionalization, heteroatom doping, and heterogeneous structure construction [70, 71, 72].

### 3.1 Surface Functionalization

The surface of GQDs is rich in oxygen‐containing functional groups, including hydroxyl and epoxy moieties, which serve as active sites for further chemical modification, along with hydrophobic π structures. Surface functionalization refers to the process of introducing or modifying specific functional groups on the surface of GQDs through methods such as oxidation, polymer grafting, or chemical group modification (e.g., carboxyl, amino groups), to alter their surface chemical properties and physical behaviors such as carrier dissipation. This process aims to regulate the interface characteristics of GQDs, thereby imparting or enhancing specific functions without disrupting their core carbon backbone structure [73, 74, 75].

Gomes et al. covalently grafted GQDs onto carbon fibers (CF) via ester bonds under low‐temperature conditions, successfully constructing a covalent interface between GQDs and CF, which solved the problem of GQD aggregation. The study showed that GQDs were uniformly distributed on the CF surface, and after grafting, the surface defects of CF increased, which promoted ion diffusion and charge storage. When the GQD‐CF composite was pressed into a flexible fabric‐like structure and used as an electrode in a symmetric supercapacitor, its specific capacitance reached 211 F/g (compared to 40 F/g for CF alone), representing an enhancement of approximately 5.5 times. The supercapacitor retained around 97% of its capacitance after 5000 cycles, and it maintained stable performance even under bending [76]. Such interface‐engineering methods improve the dispersibility of GQDs and have stimulated the development of other green and controllable surface‐modification strategies. Li et al. proposed a simple, rapid, and environmentally friendly post‐oxidation treatment for GQDs. Through a hydrogen peroxide–driven catalytic reaction in an ionic liquid, they achieved precise regulation of epoxy groups on the GQD surface. This modification method exhibits high selectivity and can significantly increase the surface epoxy group content (XPS shows the C–O–C fraction increasing from 21.42% to 29.94%) while maintaining the original size and concentration of the GQDs, thereby enhancing their surface chemical reactivity [77]. Researchers have explored the introduction of other types of functional groups, beyond simply increasing the content of oxygen‐containing moieties, to further expand the chemical tunability of GQDs. Iannazzo et al. performed an eco‐friendly surface functionalization of GQDs via a 1,3‐dipolar cycloaddition reaction conducted in a natural deep eutectic solvent (NADES). NADES, composed of betaine and glycolic acid, was employed as the reaction medium, enabling two nitrones to undergo a cycloaddition reaction with the π‐electron cloud on the surface of GQDs under microwave assistance. This strategy successfully covalently anchored ester and phosphonate groups onto the QDs. The work represents the first realization of a direct cycloaddition reaction on the π‐cloud of GQDs, providing a new avenue for surface modification of GQDs [78]. To address the issue of excessive oxygen vacancies in indium oxide (In_2_O_3_)‐based thin‐film transistors (TFTs), which typically leads to performance degradation, Gao et al. developed GQD‐modified In_2_O_3_/ZrO_2_ TFTs using a solution‐processed fabrication approach. The resulting devices achieved outstanding electrical performance and low operating voltage, including a field‐effect mobility as high as 34.02 cm^2^/V·s, a high on/off ratio of 4.55 × 10^7^, a low subthreshold swing of 0.08 V/dec, and excellent stability under both positive and negative bias stress. In the device structure, ZrO_2_ serves as the gate dielectric, and GQDs‐In_2_O_3_ forms the channel layer. The study demonstrates that this fabrication strategy enables devices with good electrical uniformity and low noise characteristics [79].

### 3.2 Heteroatom Doping

**FIGURE 3:**
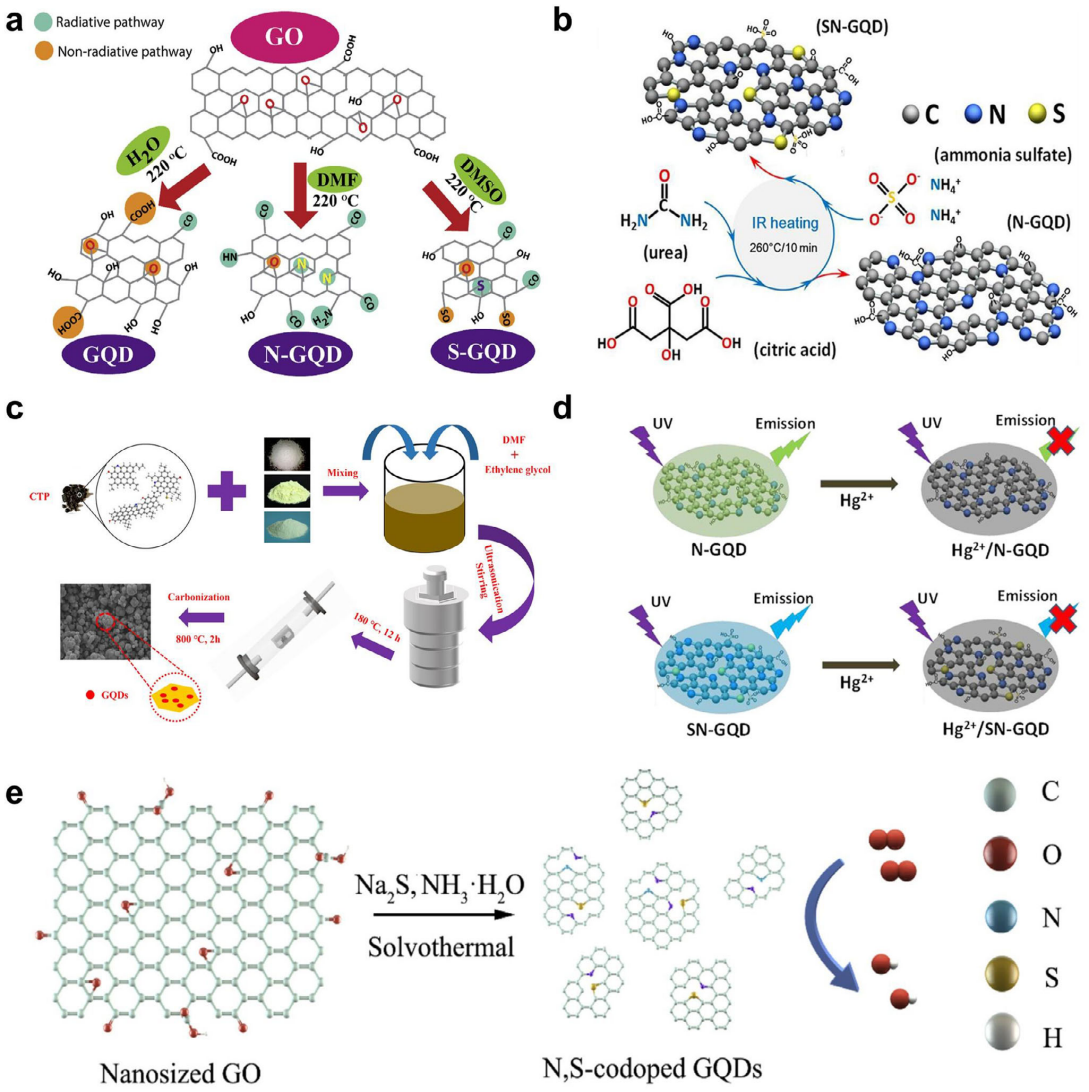
Modification strategies of GQDs. a) Reaction scheme for the synthesis of various types of GQDs using different solvents. Reproduced with permission [88]. Copyright 2020, Elsevier. b) Schematic illustration of the growth process for N‐GQD and SN‐GQD samples via an IR‐assisted technique. Reproduced with permission [80]. Copyright 2019, American Chemical Society. c) Proposed schematic representation of FeS@GQDs composite formation. Reproduced with permission [97]. Copyright 2022, American Chemical Society. d) Fluorescence quenching test of N‐GQD and SN‐GQD samples in the presence of Hg^2+^ ions. Reproduced with permission [80]. Copyright 2019, American Chemical Society. e) Illustration of the one‐pot synthesis of N,S‐GQDs. Reproduced with permission [96]. Copyright 2019, Elsevier.

Heteroatom doping refers to the incorporation of non‐carbon elements, such as nitrogen, chlorine, sulfur, or phosphorus, into the carbon lattice of GQDs through chemical methods. This process enables the regulation of their electronic configuration, band structure, and surface chemical properties. This process modifies the physicochemical characteristics of GQDs in a targeted manner by substituting carbon atoms or forming surface functional groups. Doping can introduce additional active sites, alter the bandgap and charge distribution of GQDs, thereby tuning their optical absorption wavelength (e.g., redshift or blueshift), enhancing photoluminescence, and increasing the fluorescence quantum yield. However, the doping process requires precise control over the elemental ratio, bonding configuration, and spatial distribution; otherwise, it may lead to inconsistent performance or undesired side reactions. Certain heteroatoms (such as phosphorus and sulfur) can also form defect states or surface functional groups that act as nonradiative recombination centers, causing fluorescence quenching. High doping concentrations may induce lattice distortion or the accumulation of surface defects (e.g., phosphorus doping tends to generate the highest defect density), ultimately compromising material stability and reproducibility. At present, mainstream doping strategies include single‐element doping and co‐doping (Figure 3b,d) [80, 81, 82, 83, 84].

Nitrogen doping is typically achieved using ammonium hydroxide, urea, and other nitrogen‐containing precursors, and is the most common doping strategy [85, 86, 87]. When nitrogen atoms substitute carbon atoms, they provide additional electrons, raising the Fermi level, narrowing the bandgap, and often inducing a redshift in fluorescence emission. This substitution also significantly enhances electrical conductivity and electrocatalytic activity. Giri et al. synthesized N‐GQDs via a solvothermal method (Figure 3a) and systematically investigated the mechanisms underlying their high photoluminescence quantum yield and strong surface‐enhanced Raman scattering (SERS) sensitivity. The study revealed that N‐GQDs synthesized in DMF exhibit a fluorescence quantum yield of up to 34%, attributed to the electron‐donating characteristics of nitrogen dopants and the reduced formation of nonradiative recombination centers (such as carboxyl groups) during synthesis. Furthermore, by combining N‐GQDs with RhB, white‐light emission with CIE coordinates of (0.30, 0.34) was successfully achieved [88]. Gong et al. developed an environmentally friendly, top‐down synthetic approach using low‐cost graphite as the precursor and eliminating the need for strong acids, successfully producing blue‐ and green‐emitting N‐GQDs. In this strategy, ammonium persulfate was innovatively employed as both the oxidizing agent and nitrogen source, and a dichloromethane–water solvent extraction method was used for purification for the first time, enabling the separation of two types of N‐GQDs with distinct sizes and surface chemistries. The overall yield reached as high as 52%. The resulting N‐GQDs exhibited high photoluminescence quantum yields (up to 63.8% for the green‐emitting product), excellent photostability, and low cytotoxicity [89].

Sulfur doping modifies the electronic structure and band characteristics of GQDs through the incorporation of sulfur atoms, thereby tuning their optical, electrochemical, and catalytic properties. Sulfur atoms are typically introduced into the carbon framework in the form of C–S bonds or structures such as C_SO_2_–C, and may reside either within the lattice or at edge sites. However, sulfur doping often introduces a relatively high density of structural defects (as evidenced by significantly increased defect signatures in Raman spectra). These defects can generate quenching centers, resulting in a slight decrease in photoluminescence intensity. Kim et al. synthesized sulfur‐doped GQDs (S‐GQDs) via a one‐step pulsed laser ablation in liquid (PLAL) technique, using graphite as the carbon source and 3‐mercaptopropionic acid (MPA) as the sulfur precursor in an ethanol medium. Under laser‐induced high‐temperature and high‐pressure conditions, effective sulfur incorporation was achieved. The resulting S‐GQDs showed markedly enhanced UV absorption, and their fluorescence quantum yield increased from 0.8% (pristine GQDs) to 3.89% at 10 mol% sulfur doping, while also exhibiting excellent photostability. Time‐resolved photoluminescence analysis revealed that the improved optical properties arise from sulfur doping, promoting the radiative recombination of intrinsic states in the carbon lattice [90]. Wang et al. developed a simple hydrothermal method to prepare highly luminescent S‐GQDs and successfully tuned their photoluminescence properties by adjusting the amount of sulfur powder used for doping. The results show that sulfur is incorporated in two configurations: thiophene‐like sulfur and oxidized sulfur. The incorporation of sulfur‐related energy levels alters the electronic structure of the material, which plays a crucial role in enhancing its optical properties [91].

Phosphorus doping introduces phosphorus atoms, which have a relatively large atomic radius and low electronegativity, and significantly alters the electronic structure and defect states of GQDs. Its modification effects are typically manifested as pronounced fluorescence quenching and enhanced electrochemical activity. Based on this doping mechanism, Ma et al. synthesized phosphorus‐doped GQDs (P‐GQDs) with a high phosphorus content (47 at%) through an electrochemical approach. The results indicate that phosphorus doping generates a large number of structural defects, and that phosphorus atoms function as active sites by forming structures such as C_3_PO and C_2_PO_2_/CPO_3_. Through reactions with free radicals to form additional P–O bonds or through reduction by DPPH, these sites can efficiently scavenge free radicals [92]. In addition, phosphorus doping shows substantial potential in the field of photoelectric conversion. Liu et al. modulated the electronic properties of GQDs through phosphorus doping and constructed an efficient metal‐free photocatalyst. The formation of the resulting p‐n junction structure effectively promoted the separation of photogenerated electron‐hole pairs, thereby significantly enhancing the photocatalytic performance under visible light irradiation [93]. To improve doping efficiency while maintaining a green synthesis strategy, Ding et al. developed an environmentally friendly and efficient method for producing lattice‐P‐GQDs using soy lecithin (a phosphorus‐containing compound) as the precursor. The resulting P‐GQDs exhibited a high yield of up to 71 wt% and demonstrated excellent tunable photoluminescence, with emission wavelengths spanning the visible range from 457 to 632 nm. Their quantum yields reached 0.54–0.73, and the materials showed strong photostability and resistance to ionic interference [94].

Co‐doping involves incorporating two or more heteroatoms into the carbon framework or edge sites of GQDs through chemical synthesis methods such as hydrothermal or electrochemical approaches. These atoms form stable chemical bonds (e.g., C–N, C–S, P═O) and may generate new doping‐induced energy levels or defect configurations. Different dopant atoms can complement each other in tuning the band structure such as narrowing the bandgap or introducing intermediate energy levels, thereby enhancing light absorption or charge‐separation efficiency. Single‐element doping typically improves only one specific property (for example, enhancing fluorescence or increasing conductivity), whereas co‐doping leverages the synergistic effects between multiple dopants to achieve functionalities unattainable by single doping alone. However, this approach requires precise control over dopant ratios and spatial distribution (edge vs. basal plane); otherwise, phase separation or structural disorder may occur. Dopant combinations with large atomic‐radius differences (such as P and N) may also intensify lattice strain and cause irreversible structural damage. Roy et al. synthesized Ag nanoparticle–S‐GQD nanocomposites through a one‐step method and systematically investigated their synergistic antibacterial activity and cytocompatibility. In this Ag@S‐GQDs nanocomposite, S‐GQDs served as both the reducing agent and stabilizer, enabling the uniform dispersion and stable presence of AgNPs [95]. In addition to synergistic enhancement in antibacterial applications, co‐doping can also markedly improve electrocatalytic activity. Min et al. developed a simple hydrothermal synthesis method using ammonium hydroxide (NH_4_OH) and sodium sulfide (Na_2_S) as precursors to prepare nitrogen–sulfur co‐doped GQDs (N,S‐GQDs) (Figure 3e). Through a comparative analysis of the structure and performance of undoped GQDs, singly doped samples (N‐GQDs and S‐GQDs), and co‐doped samples (N,S‐GQDs), it was found that the N,S‐GQDs exhibited a high nitrogen doping level of 9.36% and a sulfur doping level of 0.78%. The unique defect structures and the synergistic interaction between N and S significantly enhanced the oxygen reduction reaction (ORR) catalytic activity [96]. Further integrating co‐doped systems with inorganic components to construct composite structures, Zhang et al. used coal tar pitch as the precursor for GQDs and synthesized FeS@GQDs composites through a low‐cost and environmentally friendly one‐step solvothermal method (Figure 3c). The results showed that FeS and GQDs form strong coupling through C–S–C bonds, which not only provides a large specific surface area and a well‐defined porous structure, but also enhances electronic conductivity and structural stability [97].

### 3.3 Heterostructure Construction

Heterostructure construction refers to the integration of GQDs with other materials such as semiconductors, metals, polymers, or two‐dimensional materials, through physical or chemical methods to form composite structures with interfacial synergistic effects. Through mechanisms such as interfacial charge transfer, band structure modulation, and an increase in active sites, these heterostructures can exhibit significantly improved performance compared with their individual components, achieving functional complementarity and overall performance enhancement. However, this approach requires precise control over the interfacial bonding modes (e.g., covalent bonding, electrostatic interactions, van der Waals forces) and faces challenges such as synthetic complexity and potential interfacial degradation caused by mismatches in thermal expansion coefficients or differences in chemical stability between the combined materials [98, 99, 100].

Zheng et al. successfully prepared GQDs‐modified Bi_2_WO_6_ composites using a microwave‐assisted hydrothermal method. By controlling the reflux temperature and the initial concentration of reduced graphene oxide (rGO), they achieved precise tuning of GQD size (2.2–5.2 nm), thereby overcoming the limitations of traditional methods that are time‐consuming and yield poorly uniform particle sizes. The study showed that GQDs establish a new electron‐transfer pathway from the Bi 6p and O 2p orbitals of BWO to the GQDs, effectively promoting the separation of photogenerated charge carriers. Moreover, smaller GQD sizes further enhance visible‐light absorption and improve charge‐separation efficiency [101]. Liu et al. prepared two sheet‐like nano‐heterostructure photocatalysts, Sn_3_O_4_/rGO and Sn_3_O_4_/GQD, using a microwave‐assisted hydrothermal method, and systematically investigated how different assembly modes influence photocatalytic performance. Their results revealed that GQDs, acting as electron acceptors, were uniformly distributed across the Sn_3_O_4_ surface, creating more efficient charge‐separation pathways. Moreover, the photosensitization effect of GQDs broadened the visible‐light response range. Utilizing GQDs as active interfacial modifiers, instead of relying on macroscopic rGO as a conductive substrate, proved to be more effective in promoting carrier separation through interface engineering [102]. Yan et al. synthesized N‐GQDs and hierarchical Bi_2_Ti_2_O_7_ microspheres via hydrothermal methods, and subsequently constructed N‐GQD/Bi_2_Ti_2_O_7_ (NG‐BIT) composites through ultrasonic dispersion and hydrothermal assembly. The study demonstrated that N‐GQDs act as electron‐trapping centers through their nitrogen atoms, efficiently capturing and transferring the electrons generated in Bi_2_Ti_2_O_7_ upon light excitation, thereby significantly suppressing electron–hole recombination and enhancing visible‐light absorption [103]. Li et al. synthesized rose‐like hollow (BiO)_2_CO_3_ microspheres via a hydrothermal method and employed electrostatic self‐assembly to load S,N‐GQDs onto both the surface and interior of the microspheres, forming S,N‐GQDs/BOC composites. The results demonstrated that S,N‐GQDs act as charge‐transfer centers, enhancing the separation efficiency of photogenerated electron‐hole pairs. Furthermore, they accelerated the decomposition of H_2_O_2_ to generate more ·OH reactive species while mitigating the negative effects associated with excessive H_2_O_2_ accumulation during the photocatalytic process [104].

## 4 Regulation of Graphene‐Based QDs on the Working Mechanism of Neuromorphic Devices

**FIGURE 4:**
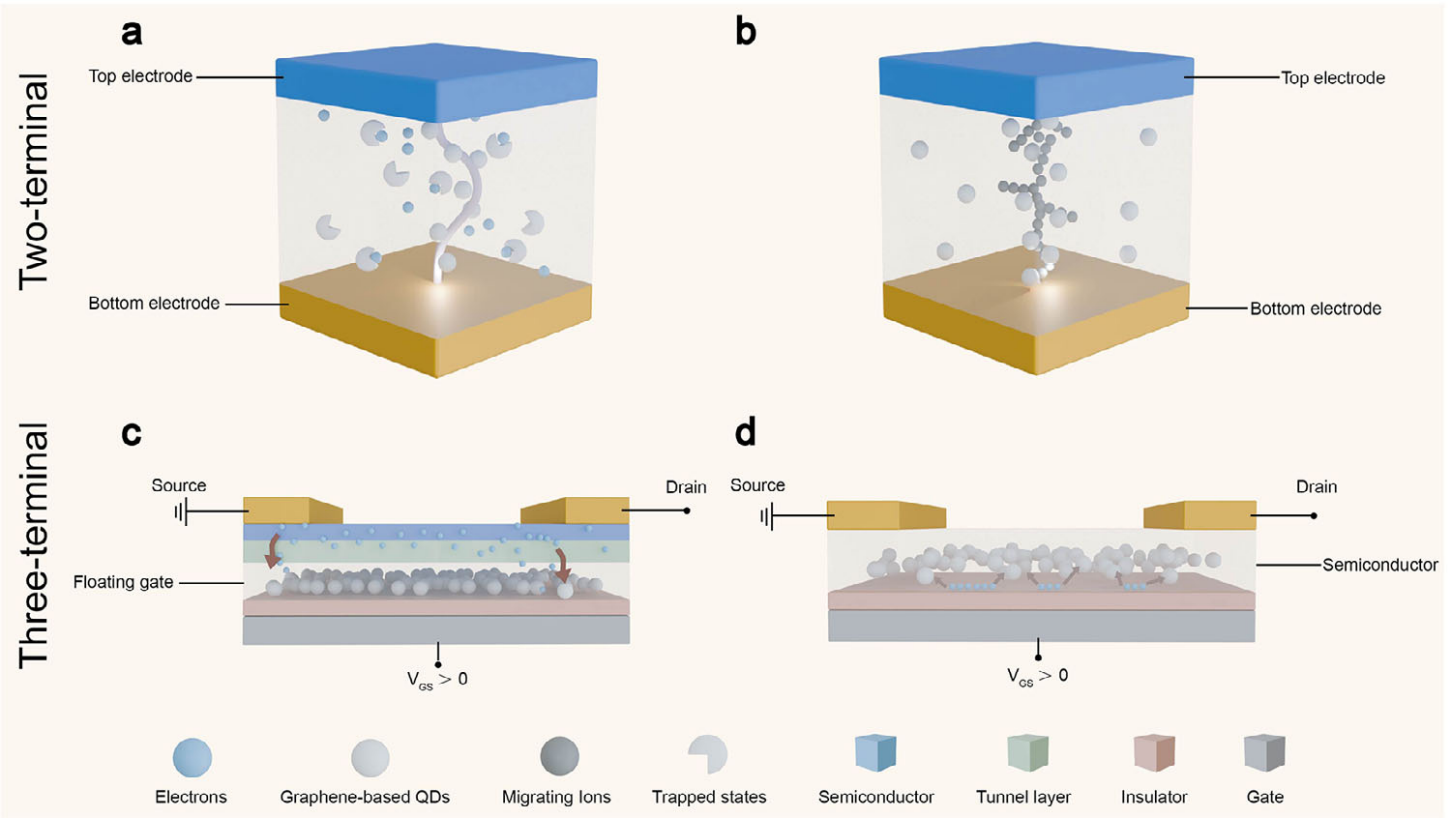
Working mechanisms of two representative two‐terminal devices: a) charge‐trapping‐type and b) ion‐migration‐type. Typical operating mechanisms of three‐terminal devices, in which graphene‐based quantum dots are incorporated into c) the floating‐gate layer and d) the channel layer, respectively.

From the perspectives of device architecture and signal modulation schemes, neuromorphic devices based on graphene‐based QDs can be broadly categorized into two‐terminal and three‐terminal configurations, which exhibit fundamentally distinct operating modes and regulatory mechanisms. Two‐terminal devices are predominantly memristors, typically adopting a metal–insulator–metal (MIM) structure, in which synaptic weight modulation relies mainly on current‐driven internal state evolution processes, including charge trapping/detrapping dynamics as well as electric‐field‐induced ion migration and redox reactions. In such devices, GQDs or GOQDs are commonly incorporated into the active layer or interfacial regions as discrete charge‐trapping centers, ion‐migration regulation nodes, or conductive filament nucleation sites, thereby effectively suppressing stochasticity and enabling controllable resistive switching behavior with continuous multilevel conductance modulation (Figure 4a,b).

In contrast, three‐terminal devices introduce a gate electrode to achieve electric‐field‐driven modulation of channel conductance, thereby realizing read–write separation at the physical level. In these devices, graphene‐based QDs can function as interfacial defect passivation and regulation units, floating‐gate charge storage nodes, or functional modifiers of the channel, significantly enhancing the linearity, symmetry, and energy efficiency of weight updates (Figure 4c,d). Moreover, benefiting from their size‐dependent optical absorption, tunable band structure, and abundant surface states, graphene‐based QDs can be further employed in optoelectronic synergistic neuromorphic devices, where optical stimuli directly participate in charge trapping or ionic transport processes. Overall, across different device configurations, GQDs govern charge trapping, ion migration, and photogenerated carrier‐involved processes through differentiated regulation of trap energy level distributions, local electric fields, and carrier/ion transport pathways, thereby enabling the synergistic optimization of weight modulation accuracy, operational stability, and energy consumption in diverse neuromorphic devices.

### 4.1 Two‐Terminal Charge Trapping Devices

Two‐terminal charge trapping devices are based on external stimulation to regulate carriers in localized traps to achieve non‐volatile resistance switching. Under forward bias, carriers tunnel and are injected into deep‐level traps. When their occupancy reaches a critical threshold, an extended conductive pathway is established, resulting in the device transitioning from a high‐resistance state to a low‐resistance state [105]. Therefore, the trap barrier depth can be controlled through band engineering, allowing the captured charge to be retained for a long time even under zero‐field conditions. Under reverse bias excitation, the trap releases charges through field‐induced de‐trapping or carrier recombination mechanisms, restoring the device to its initial high‐resistance state, thus completing the full write/erase cycle [106]. However, the key to achieving high‐performance devices lies in the precise coordination of trap density, distribution, and energy levels to simultaneously achieve a high switching ratio, excellent retention characteristics, and cycling stability. Traditional single dielectric materials face difficulties in effectively regulating this, and performance enhancement is subject to bottlenecks. To address this issue, GQDs, as a type of nanomaterial with excellent regulatory capabilities, provide a new path for the precise design of charge trapping behavior. With their quantum confinement effect and abundant surface functional groups, GQDs can introduce deep trap sites with uniform spatial distribution and precise energy level structures in composite media, effectively regulating the dynamics of charge storage and release.

**FIGURE 5:**
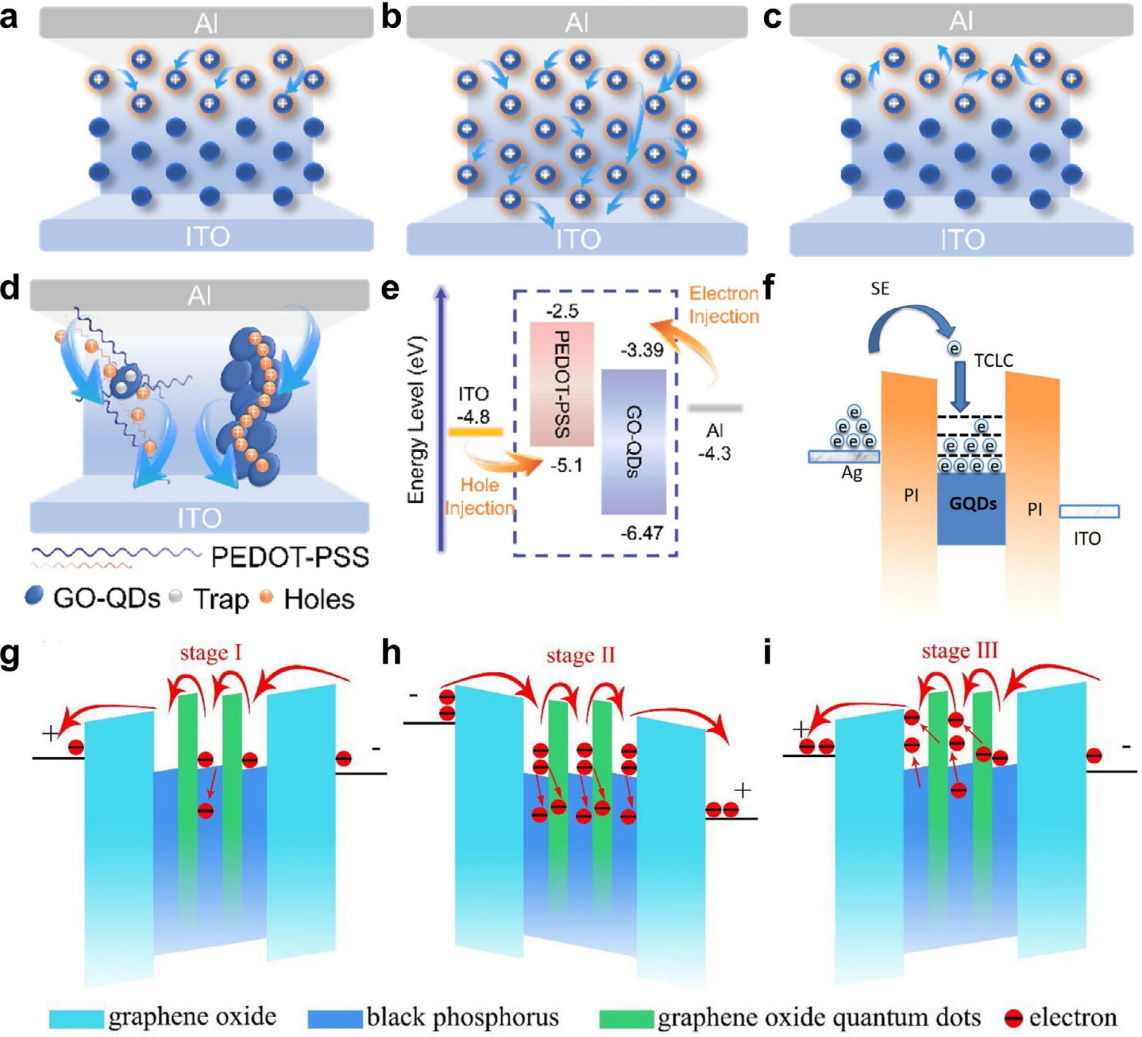
Charge‐trapping mechanisms in two‐terminal devices. a) Schematic representation of the charge capture stage. b) Illustration of the trap filling phase. c) Depiction of the charge detrapping process. d) Schematic diagram of hole transfer. e) Energy level structure diagram. Reproduced with permission [107]. Copyright 2025, Elsevier. f) Possible charge transportation occurred through the fabricated memristor device under the applied. Reproduced with permission [108]. Copyright 2023, Springer Nature. g) Initial state I of the device under 0.01 V bias. h) State II under −2 V pulse voltage, where a large number of electrons are injected into the BP·GOQD layer, with some electrons directly reaching the ITO electrode. i) State III after the −2 V pulse, where some electrons trapped in the BP·GOQD layer can separate from holes and return to the Ag electrode under a 0.01 V bias. Reproduced with permission [109]. Copyright 2022, American Chemical Society.

To explore the resistance switching mechanism of quantum dot composite films doped with organic materials, Qu et al. used a solution spin‐coating method to fabricate high‐performance organic memristors based on GOQD‐doped PEDOT:PSS composite films. The device exhibited optimal performance at a 60% GOQD doping concentration, achieving a switching ratio (I_on_/I_off_) as high as 10^4^ and long‐term data retention exceeding 10^4^. They also confirmed that the resistance switching mechanism was derived from the charge trapping and release processes induced by PEDOT:PSS or GOQDs (Figure 5a‐c). GOQDs, with their surface‐rich oxygen‐containing functional groups and inherent lattice defects, can form deeper trap energy levels by aggregating into charge trapping centers, capturing more hole carriers (Figure 5d,e). As the concentration of GOQDs increases, these aggregates become interconnected, facilitating efficient charge transport between neighboring QDs [107]. Utilizing the ability of GQDs to form uniformly distributed charge traps in polymer matrices, Li et al. used the spin‐coating technique to fabricate memristive synaptic devices based on polyimide (PI) and GQDs composites (Ag/PI:GQDs/ITO). The study demonstrated that the thermionic emission current observed at low voltages is primarily based on thermally excited electrons. Under forward bias, electrons can overcome the PI barrier and are more easily captured into the rich energy level structure of GQDs (Figure 5f), at which point the device exhibits a high‐conductance state. As more electrons are rapidly captured by the trap centers formed by the uniformly distributed GQDs, the establishment of an internal electric field drives the device to transition to a low‐conductance state. When a reverse bias voltage is applied, the captured electrons can be released from the traps, and the internal electric field is disrupted, restoring the device's conductance to the high‐conductance state. By controlling the pulse amplitude and frequency, the amount of charge injected into the device can be adjusted, demonstrating plasticity characteristics that depend on both amplitude and frequency [108]. To enhance the stability and electronic storage capacity of two‐dimensional black phosphorus (BP), Wei et al. employed a strategy of compositing GOQDs with BP to fabricate a stable and low‐power artificial synaptic device based on the BP‐GOQD heterostructure. The study showed that the introduction of GOQDs generates discrete energy levels, forming electron capture barriers that limit electron transport and enhance storage capability. When a voltage pulse is applied, GOQDs capture some of the injected electrons (Figure 5g,h), and the remaining electrons form a current through the BP layer. After the pulse ends, the captured electrons are slowly released (Figure 5i), generating sustained postsynaptic currents that simulate the conversion from short‐term memory (STM) to long‐term memory (LTM) in biological synapses. This study addressed the issue of declining electronic storage capacity with cycling in pure BP devices through the synergistic electron trapping of BP‐GOQD [109].

Based on the aforementioned studies, the significant quantum confinement effect and good band alignment of GQDs enable the construction of deep trap networks with precise energy levels and spatial distribution in various media systems. Moreover, the charge trapping layer based on GOQDs can form deeper trap energy levels, which helps achieve stable charge capture, allowing the device to retain its resistance state for an extended period even after the external electric field is removed. This provides a reliable physical foundation for high‐performance non‐volatile memory.

### 4.2 Two‐Terminal Ion Migration‐Based Devices

Two‐terminal ion migration‐based devices primarily rely on the migration of metal cations (such as Ag^+^, Cu^2+^) or anions (such as O^2−^) under external stimuli (such as electric fields, temperature gradients, or chemical potential gradients) and redox reactions to govern the formation and rupture of conductive filaments (CFs), thereby controlling the resistance switching behavior [110]. The composition, morphology, and stability of the CFs can all influence the device's durability and the precision of resistance value control. However, due to various uncontrollable factors (such as lattice defects in the active layer, uneven electric field distribution, etc.), the uncontrollable diffusion of metal ions is often accompanied by the random growth of CFs, which severely affects the reliability and uniformity of the device's performance [111].

#### 4.2.1 Electrochemical Metallization Devices

**FIGURE 6:**
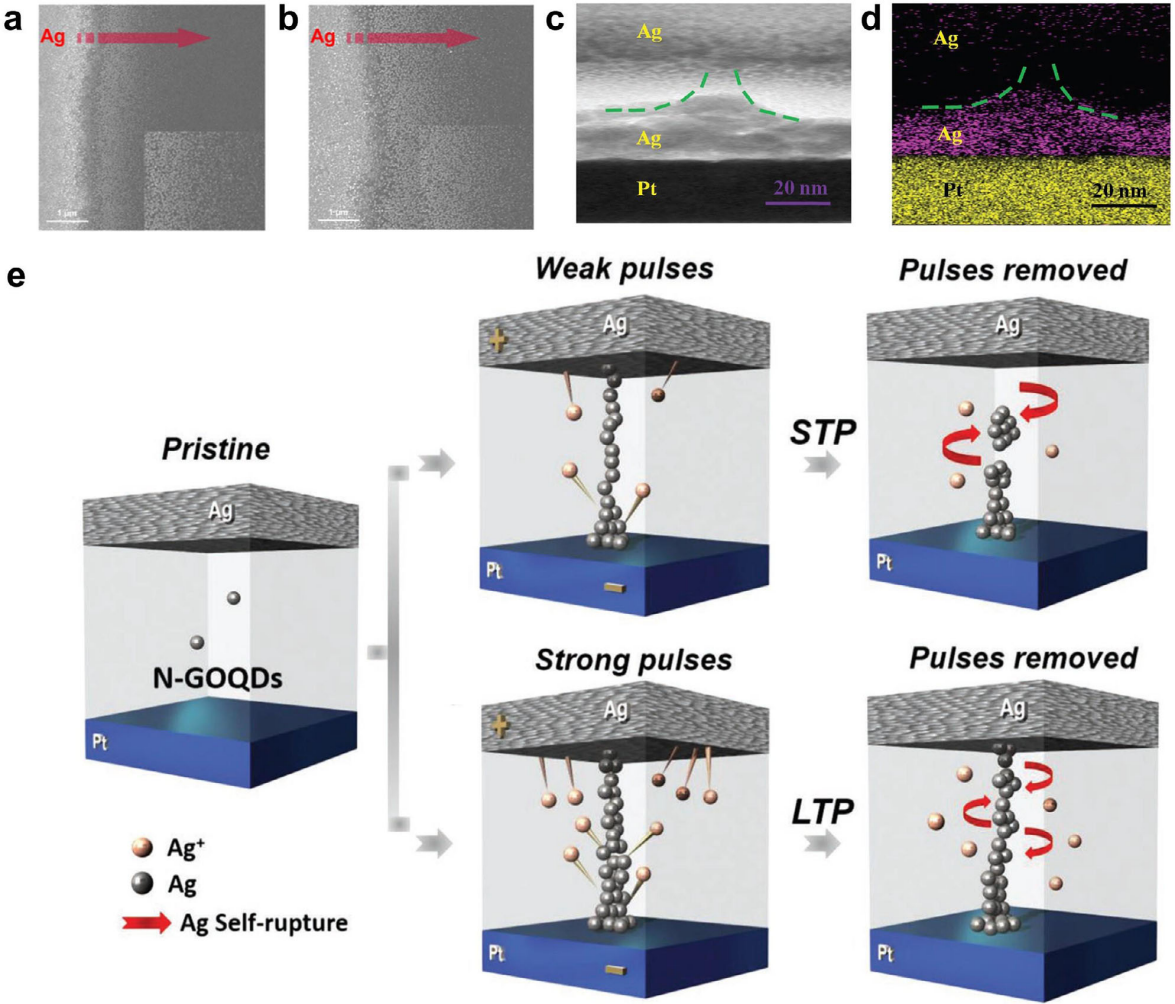
Ion‐migration mechanisms in two‐terminal devices. a, b) Formation mechanism of Ag cluster‐type conductive filaments in an Ag/PVP–GOQD/Ag planar device as the applied voltage increases. Reproduced with permission [112]. Copyright 2024, American Chemical Society. c) TEM image of the AZA device. d) Mapping image of the AZA device. Reproduced with permission [113]. Copyright 2018, John Wiley and Sons. e) Mechanism of Ag^+^ cation migration‐induced switching in N‐GOQD‐based synaptic devices. Reproduced with permission [114]. Copyright 2019, John Wiley and Sons.

To suppress the randomness in the ion migration process, control the growth of CFs, and enhance device performance, GOQDs can be introduced into functional media to act as nucleation sites for the migration and reduction processes of metal ions, enabling spatial and energy control. Kim et al. doped GOQDs into a polyvinylpyrrolidone (PVP) matrix to construct a synaptic device based on a PVP/GOQD nanocomposite material with guided filament growth characteristics. In this device, GOQDs act as nucleation centers for the reduction of silver ions, owing to their high specific surface area and abundant surface functional groups. When the device is subjected to positive pulse stimulation, Ag+ migrates from the Ag electrode to the active layer, where, under the influence of electron capture by the GOQDs, Ag^+^ is preferentially reduced and aggregated around the GOQDs, ultimately forming a directional cluster of CFs from the anode to the cathode along the electric field direction (Figure 6a,b) [112]. To further improve the device's stability and uniformity, Zhou et al. introduced GOQDs into Zr_0.5_Hf_0.5_O_2_ to construct a memristor device with an Ag/Zr_0.5_Hf_0.5_O_2_:GOQDs/Ag structure. In this system, GOQDs, as an interface modification layer, enhance the local electric field and regulate the ion migration path, enabling Ag^+^ ions to be controllably reduced to Ag atoms under the influence of the electric field, and form discontinuous Ag nanoclusters with controllable gaps within the ZHO (Figure 6c,d). The inherent quantum confinement effect and abundant surface states of GOQDs synergistically modulate the migration of Ag^+^ ions and the interface charge behaviors, including Fowler‐Nordheim tunneling and direct tunneling. This enables bidirectional progressive linear conductance modulation under low‐energy pulses of 0.6 V and 30 ns [113]. Based on the high surface area and excellent insulating properties of N‐GOQDs, Choi et al. developed a novel biocompatible memristive synapse with a N‐GOQD functional layer. The study showed that when a small forward voltage is applied to the Ag electrode, a small number of Ag^+^ cations are injected at the Ag/N‐GOQD interface, forming unstable CFs that can be captured by the high‐insulation layer of N‐GOQDs. After the stimulus is removed, the device can restore its insulating properties due to the synergistic effect of surface energy minimization at the interface and the stretching stress caused by the formation of silver filaments. However, under the application of a strong positive voltage, a large amount of Ag^+^ can form stable CFs, simulating the memory transition from short‐term plasticity (STP) to LTP in biological synapses (Figure 6e) [114].

#### 4.2.2 Valence Change Devices

Hong et al. used reduced graphene oxide‐quantum dot (RGO‐GQDs) composites to fabricate a memristive synaptic device based on a cross‐array structure. The study found that when a negative bias voltage is applied to the device, O^2−^ ions migrate toward the electrode interface in the RGO‐GQDs layer, resulting in an increase in oxygen vacancies at the interface, which leads to the formation of CFs. High‐resolution transmission electron microscopy revealed that under these conditions, crystallized regions form within the RGO‐GQDs area, with a decrease in interlayer spacing, an increase in the C‐C bond ratio, and enhanced stability of the conductive channels. When a positive bias is applied, O^2−^ ions diffuse back into the RGO layer, causing the conductive filament to break, the oxygen functional groups to reform, and the resistance to increase. The edge structure of GQDs results in a significant shift of the G‐band (1518 cm^−1^) in the Raman spectrum with position, and the average size of the GQDs, calculated from the G‐band shift, is found to be between 5.8 and 8.9 nm. This size effect localizes the electric field and prevents the sudden formation of conductive channels. Additionally, the heterogeneous interface between GQDs and the uniformly thick RGO layer exhibits local coupling atomic layers, and by regulating the density of oxygen vacancy defects, the stability of the conductive channel is improved, reducing resistance fluctuation between cycles (<3%) [44].

Based on an in‐depth analysis of the growth and formation evolution mechanisms of metal CFs, the researchers of the aforementioned studies innovatively doped GQDs and their derivatives into the active layer. Their high specific surface area and uniform dispersion offer numerous active sites for ion migration and help regulate the growth direction and morphology of CFs. Additionally, the sp^2^ hybridized carbon structure and edge defects of GQDs can control the diffusion and accumulation of O^2−^, preventing the sudden formation of conductive channels. This addresses the key issue of random conductive filament growth in traditional devices and successfully improves the device's cycling durability and the precision of conductance state control.

### 4.3 Three‐Terminal Charge Trapping Devices

Compared with two‐terminal devices, three‐terminal devices enable independent gate control of channel conductance, thereby realizing read–write separation and avoiding state perturbations induced by the high read/write currents commonly encountered in two‐terminal memristors. This capability is crucial for improving the retention and reproducibility of synaptic weights in neuromorphic devices. Because the modulation mechanism is governed by an electric field rather than by the migration of a large number of charge carriers, three‐terminal devices can adjust the charge distribution, polarization state, or ionic accumulation within the channel in a more gentle and fine‐grained manner. Consequently, conductance modulation tends to be more linear and symmetric, facilitating low‐noise, multilevel, quasi‐continuous weight update behaviors that meet the training requirements of high‐precision neural networks. In addition, electric‐field‐driven write operations significantly reduce energy consumption and mitigate the risks of structural fatigue and performance degradation under frequent operation [115, 116, 117].

The introduction of functional materials simultaneously reshapes channel transport and electric‐field‐driven charge regulation pathways, thereby determining the linearity of weight updates, threshold voltage stability, and energy consumption [118]. Owing to their abundant surface states, discrete quantum energy levels, and effective passivation of interfacial defects, GQDs are capable of modulating multiple dimensions of the operating mechanisms in three‐terminal transistors, including channel transport, charge storage, and trap‐state dynamics. Gao et al. fabricated enhancement‐mode thin‐film transistors (TFTs) using ZrO_2_ as the gate dielectric and a GQDs–In_2_O_3_ composite as the channel layer. When a positive gate voltage (V_GS_) is applied, positive charges accumulate in the ZrO_2_ dielectric, inducing electron accumulation at the GQDs–In_2_O_3_ channel/dielectric interface to form a conductive channel, resulting in a pronounced increase in the source‐drain current (I_DS_) with increasing V_GS_. Under a reverse bias (V_GS_ < 0), channel carriers are depleted, and I_DS_ decreases to an extremely low level. The results demonstrate that the incorporation of GQDs significantly enhances the field‐effect mobility (µ_FE_) and carrier transport efficiency by reducing oxygen vacancies (Vo) and interfacial trap density (Dit = 5.84 × 10^11^ cm^−2^) [79]. Beyond serving as interfacial and defect‐regulation centers, GQDs can also act as charge storage nodes that directly participate in device state switching. Kim et al. employed GQDs as a charge‐trapping layer to fabricate organic nano floating‐gate memory transistors. Upon application of a positive gate voltage, the gate electric field drives holes from the pentacene channel through the polystyrene (PS) tunneling layer into the GQD layer. Owing to their size distribution (<10 nm), GQDs exhibit quantum confinement effects, giving rise to multiple discrete energy levels capable of trapping a large number of holes. The accumulation of holes in the GQDs reduces the carrier concentration in the channel, leading to a positive shift of the threshold voltage (V_TH_). When a negative gate voltage is applied, the electric‐field direction reverses, allowing the trapped holes in the GQDs to detrap via tunneling and return to the channel. After hole release from the GQDs, channel conductivity is restored and V_TH_ returns to its initial value, enabling reversible switching. The study demonstrated that the device achieves an on/off ratio as high as 10^6^, retains its current state for up to 10^4^ s, and exhibits endurance exceeding 100 cycles [119]. Furthermore, GQDs and their derivatives can serve as active layers for constructing structurally integrated memory transistors. Seo et al. fabricated rGOQD‐based TFTs and memory thin‐film transistors (rGOQD‐MTFTs) via solution processing. In these devices, p‐type rGOQDs with a nonzero bandgap act as the active layer, self‐assembled monolayer (SAM) Au nanoparticles (Au NPs) function as charge storage centers, and cross‐linked PVA serves as the dielectric layer, enabling non‐volatile memory operation through gate‐voltage modulation. When a positive gate voltage is applied, electrons in the rGOQD active layer tunnel through the PVA dielectric and are stably trapped at the Au NP surface, inducing a positive threshold voltage shift (ΔV_TH_ = 7.84 V) and establishing the “programmed” state. Under a negative gate voltage, the trapped electrons in the Au NPs detrap and return to the rGOQD layer, restoring the threshold voltage to its initial value and completing the “erase” operation. The study demonstrates that rGOQD‐based devices overcome the zero‐bandgap limitation of conventional graphene and exhibit saturation output characteristics comparable to those of traditional semiconductors [120].

### 4.4 Two‐Terminal and Three‐Terminal Optoelectronic Devices

Optoelectronic devices are an important branch of neuromorphic devices, combining the advantages of photonics and electronics. These devices can utilize the synergistic effect of optical and electrical signals to achieve multiple functions, such as light sensing, signal processing, and storage, within a single device [121, 122]. Their working mechanism is typically based on the photoelectric effect, where light stimulation alters the electrical state of the device (such as resistance, capacitance, etc.), thereby simulating the plasticity of biological synapses or the spike firing behavior of neurons [123, 124].

**FIGURE 7:**
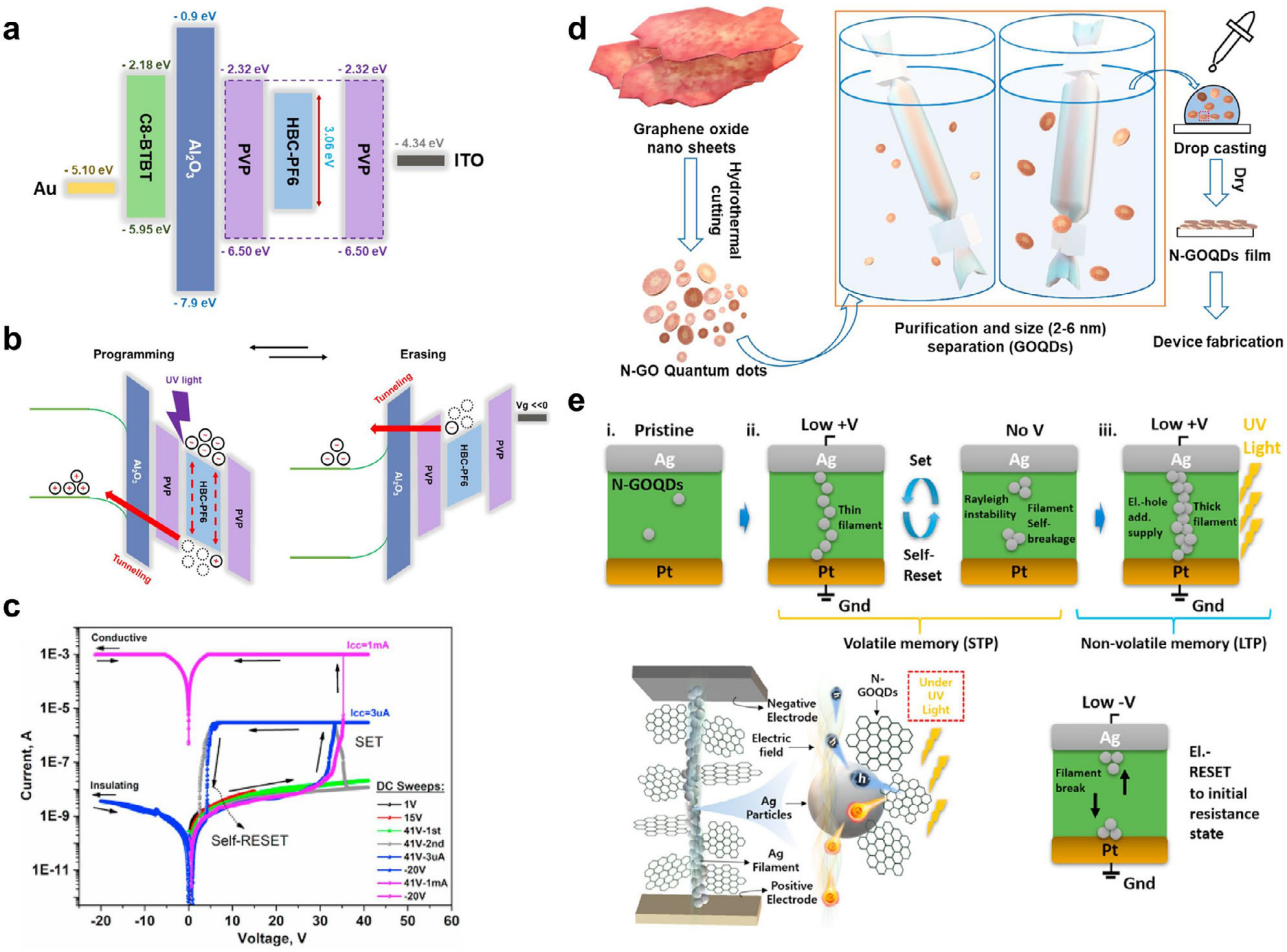
Optoelectronic neuromorphic mechanisms. a) Energy level diagrams of the individual components of the photonic synapse transistor prior to contact. b) Energy band diagrams of the photonic synapse components: under light illumination during the photo‐programming mode (left), and under negative bias during the electrical erasing mode (right), both at thermal equilibrium. Reproduced with permission [125]. Copyright 2025, American Chemical Society. c) *I–V* curves observed during in‐situ Ag filament formation. d) Synthesis of GOQDs films by the hydrothermal cutting process of graphene oxide nano‐sheets. e) Elaboration on UV illumination assisted TS mechanism. Reproduced with permission [127]. Copyright 2021, Elsevier.

To address the limitations of limited UV light responsiveness and uneven interfaces when using light‐absorbing QDs in floating gates, Park et al. employed a scheme using functionalized hexabenzocoronene‐derived GQDs as the floating gate and C8‐BTBT as the organic semiconductor channel (Figure 7a) to fabricate an ultraviolet (UV)‐responsive optoelectronic synaptic transistor. Under UV light irradiation, GQDs, with their strong absorption characteristics in the 300‐425 nm wavelength range (peaking at around 365 nm), can selectively capture UV photons and trigger effective electron‐hole pair separation. Photogenerated holes tunnel into the organic semiconductor channel, thereby increasing the conduction current. Simultaneously, electrons are trapped in the GQD floating gate, resulting in a positive shift of the threshold voltage and emulating the enhancement behavior observed in biological synapses (Figure 7b). This device simultaneously senses optical inputs and performs neuromorphic signal processing, demonstrating robust optical programming and electrical erasure characteristics [125].

Park et al. fabricated bottom‐contact pentacene/GQDs field‐effect transistors (FETs) using photolithography. In this device, a heavily doped p‐type silicon substrate serves as the gate electrode, SiO_2_ as the gate dielectric, and Au/Ti as the source and drain electrodes. In the dark state, when a positive gate voltage is applied, the gate electric field is directed toward the pentacene layer, driving electrons from the highest occupied molecular orbital (HOMO) or near‐HOMO localized states in pentacene to transfer via localized states into bandgap trap states in the GQD layer. After electron trapping in the GQD layer, hole accumulation is induced near the pentacene interface due to charge balance effects, resulting in a positive shift of the threshold voltage (V_TH_). During reverse gate‐voltage sweeping, the negative gate electric field causes the trapped electrons in the GQD layer to detrap, reducing the hole concentration and shifting the threshold voltage toward more negative values. Under illumination (650 nm laser), pentacene absorbs photons to generate singlet excitons, which undergo fission to form triplet excitons and subsequently dissociate into free electrons and holes. The gate electric field drives the electrons toward the GQD layer, while the additional photogenerated electrons further increase the amount of trapped charge in the GQDs, significantly enhancing the hole concentration in the pentacene layer and leading to a further positive shift of the threshold voltage during forward scanning. The study demonstrates that, by tuning the gate‐voltage polarity and scanning range, the device can switch between photovoltaic response and optoelectronic memory functionalities [126].

To further investigate the impact of light assistance on memristive behavior, Choi et al. used N‐GOQDs as the photosensitive ion conductor layer to construct a UV‐controlled two‐terminal diffusion‐type memristor (Figure 7d). Under UV light irradiation, the device achieved light‐assisted regulation of Ag^+^ ion migration and threshold resistance switching characteristics based on the n‐π* electron transition of N‐GOQDs and nitrogen doping‐induced synergistic enhancement of additional conductivity (Figure 7c,e). This successfully simulated various synaptic plasticity behaviors, including light‐enhanced postsynaptic current and the enhanced transition from short‐term memory to long‐term memory. The structural mechanism relies on the similarity in dynamics between Ag^+^ diffusion and Ca^2+^ biological migration triggered by UV, enabling the light modulation reproduction of heteroplastic phenomena [127].

## 5 Applications of Graphene‐Based QDs in Memory and Neuromorphic Devices

### 5.1 Impact of Surface States, Size Effects, and Heteroatom Doping on Device Performance

Key factors such as surface state characteristics, size effects, and heteroatom doping synergistically regulate the trap energy level distribution, carrier capture capability, and interfacial charge transport processes of graphene‐based quantum dots, thereby influencing the energy efficiency, electrical response, and operational stability of non‐volatile memory and neuromorphic devices [128, 129].

The surface states of graphene‐based QDs primarily originate from edge defects, dangling bonds, and oxygen‐containing functional groups (e.g., hydroxyl, carboxyl, and epoxy groups), which can establish a high‐density network of shallow and deep trap states within the dielectric layer. The surface‐state‐dominated slow charge trapping and detrapping dynamics constitute the fundamental mechanism for achieving linear and symmetric LTP/LTD in devices. Rational modulation of the trap energy depth and spatial distribution density enhances charge storage capacity while suppressing reverse tunneling processes, thereby broadening the memory window and prolonging retention time. Furthermore, oxygen‐containing groups spatially confine the migration of oxygen vacancies, stabilizing their conductive pathways, effectively reducing voltage fluctuations during resistive switching and improving cycle‐to‐cycle reproducibility [130].

The size effect directly determines the synaptic response speed and the linearity of synaptic weight modulation [131]. The quantum confinement effect induced by size reduction alters the band alignment and local electric field distribution. When the lateral dimension is confined to the few‐nanometer scale, energy level discretization is enhanced and the trap distribution becomes more uniform, thereby facilitating the realization of stable multilevel conductance states [132]. Meanwhile, the shortened carrier transport pathways and the more concentrated electric field distribution reduce the device operating voltage, thereby enabling low‐energy write operations.

Heteroatom doping enables targeted optimization of device performance through modulation of the electronic structure. Nitrogen doping introduces electron‐donating characteristics and localized defect states, thereby enhancing carrier trapping capability and increasing the switching ratio [133]. Boron doping modulates the conduction polarity, facilitating the construction of bipolar resistive switching behavior. Sulfur doping can significantly reduce the HOMO–LUMO energy gap, thereby lowering the carrier injection barrier and enhancing conductance modulation capability, enabling low‐voltage device operation [134]. Meanwhile, sulfur doping can introduce localized trap energy levels and induce slow relaxation dynamics of charges and excitons, thereby providing a critical physical basis for LTM [135]. Co‐doping can synergistically construct electron–hole dual‐trap systems, enabling more stable multibit storage and a narrower operating voltage distribution.

### 5.2 Non‐Volatile Memory

Non‐volatile memory is a type of data storage device that can retain stored data for a long period even after power is turned off. Its core feature is data persistence, meaning information can be preserved without the need for a continuous power supply. This includes traditional ROM, EPROM, widely used flash memory (such as SSDs and USB drives), and emerging technologies like MRAM and RRAM, which aim to balance speed, durability, and energy efficiency. These memory types are commonly used to store firmware, operating systems, and user data, playing a critical role in computers, mobile devices, embedded systems, and the Internet of Things (IoT), ensuring that vital information is not lost during power outages [136, 137, 138].

#### 5.2.1 Charge Trapping Memory

Charge trapping memory is a type of non‐volatile memory that utilizes nano‐scale charge trapping materials (such as metal nanocrystals or GQDs) within insulating layers (e.g., silicon nitride or alumina) to achieve data storage. Its core mechanism involves controlling the injection and release of charges through an external electric field, thereby enabling data writing, erasing, and retention. These memories offer low operating voltage, high durability, and excellent data retention capabilities (greater than 10 years at room temperature). They are widely used in applications such as NOR flash, embedded storage, and neuromorphic computing. The performance advantages of this memory are primarily reliant on the energy level control of the trapping materials and interface engineering. For instance, optimizing the size of quantum dots can achieve multi‐level storage, significantly enhancing storage density [139, 140].

Yan et al. incorporated GOQDs into the charge‐trapping layer of a high‐k HfO_2_ dielectric to fabricate a high‐performance non‐volatile memory device. This device demonstrated a broad memory window of approximately 1.57 V under a ±3.5 V voltage sweep and exhibited minimal charge loss, retaining 86.9% of its charge after 1.2 × 10^4^ s in a retention test. Following 10^5^ program/erase cycles, the memory window degraded by only 14.8%, demonstrating superior stability compared with the control device. The study revealed that the conduction band of GOQDs lies below that of HfO_2_, forming an electron quantum well that enhances charge confinement. In addition, the deep‐level defects in HfO_2_ act synergistically with the GOQDs to improve charge‐trapping efficiency, suppress electron back‐tunneling, and enhance data retention [141]. Further investigating the role of GQD doping in enhancing GO‐based resistive switching materials for multilevel data storage, Li employed a solution‐processed approach to decorate GQDs onto the surface of GO, successfully fabricating an all‐inorganic multilevel memristor (ITO/GO:0.5 wt% GQDs/Ni) exhibiting ternary resistive switching characteristics. By serving as charge‐capture centers, GQDs enabled controllable switching from binary to ternary states, yielding excellent performance: SET_1_, SET_2_, and RESET voltages of –0.9, –1.7, and 5.15 V, respectively; ON_2_/ON_1_/OFF current ratios of 10^3^:10^2^:1; a retention time exceeding 10^4^ s; and stable operation over 100 cycles. Compared with pure GO devices, the incorporation of GQDs increased the storage density by 50% and reduced the standard deviation of switching voltages across cycles. This work provides a promising device strategy for ultrahigh‐density non‐volatile memories and multibit neuromorphic computing units [142].

#### 5.2.2 Resistive Random‐Access Memory (RRAM)

RRAM, characterized by its low power consumption, high endurance, simple device architecture, holds significant promise for high‐density integration and is considered a strategic candidate for next‐generation information storage and processing technologies [143, 144]. RRAM stores data by enabling reversible switching between resistance states, which originates from the formation and rupture of CFs within the resistive switching layer [138]. Unlike conventional silicon‐based transistors, RRAM can achieve ultrahigh storage density through three‐dimensional multilayer stacking. However, challenges remain, including large variability in switching threshold voltages and issues related to device uniformity and reliability [145]. GQDs, by enhancing local electric fields and shortening ion migration pathways when incorporated into oxide matrices, can act as efficient charge‐trapping centers and conductive‐filament guiding layers, thereby improving the overall performance of RRAM devices.

**FIGURE 8:**
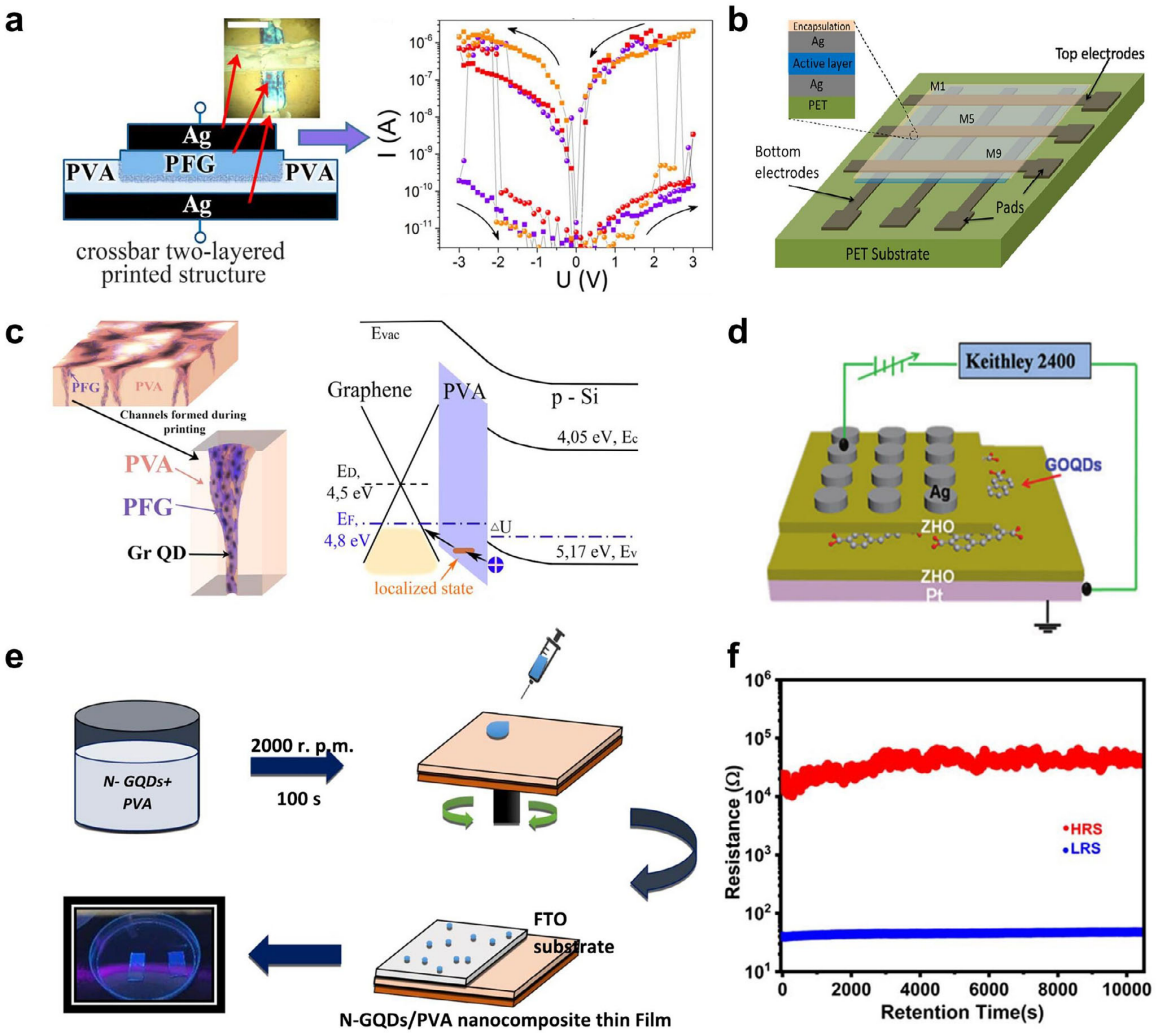
Non‐volatile memories based on graphene‐based QDs. a) Schematic illustration of the vertical PFG/PVA structure. The inset displays a photograph of the printed structure, with the graduation mark representing 1 cm. Current‐voltage characteristics of the printed vertical PFG/PVA bilayer structure with a PVA thickness of approximately 400 nm. The number of measurement cycles is indicated as a parameter, along with the volumetric ratio of the PFG/PVA suspension. Reproduced with permission [146]. Copyright 2019, IOP Publishing. b) Schematic diagram of a 3 × 3 memory array based on GQDs and PVP composite. Bottom Ag electrodes are deposited on a PET substrate, followed by the active layer of GQDs/PVP composite, and finally the top Ag electrodes. The device is encapsulated with Al_2_O_3_. The inset shows a cross‐sectional view of the structure. Reproduced with permission [147]. Copyright 2015, Elsevier. c) A quality model to describe the resistive switching effect in two‐layer films. Reproduced with permission [146]. Copyright 2019, IOP Publishing. d) Schematic of the Ag/ZHO/GOQDs/ZHO/Pt device. Reproduced with permission [148]. Copyright 2017, The Royal Society of Chemistry. e) Preparation of the N‐GQDs/PVA nanocomposite thin film using a simple spin‐coating technique. f) Retention time data for the 1 wt% N‐GQDs/PVA nanocomposite thin film measured at 0.5 V. Reproduced with permission [151]. Copyright 2024, IOP Publishing.

Threshold switching and memristive switching based on different material systems are closely related. Antonova et al. employed a two‐dimensional printing technique to fabricate a bilayer thin film composed of partially fluorinated graphene (PFG), GQDs, and PVA, achieving a resistive switching effect. In the Ag/PFG/PVA/Ag crossbar structure they constructed, stable resistive switching behavior was observed, with an ON/OFF ratio reaching 1 to 4–5 orders of magnitude (Figure 8a,c). The study further showed that decreasing the PVA thickness induces a transition from unipolar threshold switching to bipolar resistive switching. This crossbar structure maintained its performance under mechanical strain up to 6.5%, and its switching behavior remained stable for approximately one year [146]. To further investigate structure‐designed control of resistive switching, Doh et al. employed electrohydrodynamic printing to fabricate a fully printed and highly stable RRAM, utilizing a composite of GQDs and PVP on a flexible PET substrate. The device, configured as a 3×3 crossbar array (Figure 8b), demonstrated reversible switching between the high‐resistance state (HRS) and low‐resistance state (LRS), with an ON/OFF ratio of ∼14, endurance exceeding 500 switching cycles, data retention up to 30 days, and excellent flexibility withstanding bending to a radius of 8 mm. Owing to these advantages, this RRAM holds significant potential for flexible, high‐performance non‐volatile memory applications [147].

To investigate the effect of GOQDs on improving the uniformity and retention of resistive switching parameters, Chen et al. fabricated an Ag/Zr_0.5_Hf_0.5_O_2_ (ZHO)/GOQDs/ZHO/Pt stacked RRAM device by inserting GOQDs into the ZHO layer (Figure 8d). After incorporating GOQDs, the distribution ranges of the Set voltage (0.08–0.3 V) and Reset voltage (–0.14—0.01 V) were significantly narrowed, showing greatly enhanced switching uniformity compared with devices without GOQDs (Set: 0.08 – 1.25 V; Reset: –0.01—0.25 V). The power consumption for SET/RESET operations was as low as 2 × 10^−5^ and 3.9 × 10^−7^ W, outperforming GO‐ or MoS_2_‐based counterparts. The device also exhibited excellent long‐term data retention with no noticeable degradation over 1 × 10^4^ s, a stable HRS/LRS ratio, and did not require a high‐voltage forming process (forming‐free). This work offers new insights for enhancing the reliability of oxide‐based RRAM and advancing high‐performance, high‐density non‐volatile memory technologies [148]. In a study investigating the behavior of fluorinated graphene films under swift heavy ion irradiation at 167 MeV, Chernozatonskii et al. utilized high‐energy Xe‐ion irradiation to induce the formation of GQDs, measuring 1‐3 nm in size, within the fluorinated graphene matrix. The resulting material exhibited excellent electrical activity and stability, with a QD bandgap of approximately 1–1.5 eV, a dot density of (1–2) × 10^11^ cm^−2^, and characteristic QD trapping peaks observed in the C–V and G–V profiles. The researchers further applied this material to a printed FG/PVA memristor structure, achieving stable bipolar resistive switching with an ON/OFF current ratio of 10^3^–10^5^. This work establishes a controllable structural foundation for fluorinated‐graphene‐based memristors and quantum electronic devices [149]. Building on this foundation, Olejniczak et al. further employed high‐energy xenon‐ion irradiation to fabricate memristors using fluorinated‐graphene/poly(vinyl alcohol) (FG–PVA) active layers. The study revealed that irradiating the active layer (dielectric FG film/PVA) in a 2D‐printed crossbar structure enabled stable memristive switching with an ON/OFF current ratio spanning 2 to 4 orders of magnitude, switching speeds of 30‐40 ns, and excellent endurance exceeding 10^3^ cycles. Owing to its simple architecture, printability, and good mechanical flexibility, this device shows strong potential for next‐generation high‐performance non‐volatile memory applications [150]. Leveraging the non‐toxicity, thermal stability, good water solubility, and excellent film‐forming capability of N‐GQDs dispersed in PVA, Bhave et al. fabricated a degradable memristor based on an N‐GQDs/PVA nanocomposite using a simple spin‐coating process (Figure 8e). At an N‐GQD doping concentration of 1 wt%, the device exhibited optimal performance, including an ON/OFF ratio of ∼10^2^, data retention up to 10^4^ s, and cycling stability over 50 switching cycles (Figure 8f). The device also showed remarkable water‐responsive degradability, undergoing physical disintegration within 3 min of immersion. This feature enables its use as a disposable secure memory for applications in fields such as military systems and anti‐counterfeiting, where rapid destruction after use is essential to prevent data leakage [151].

### 5.3 Artificial Neuron Devices

**FIGURE 9:**
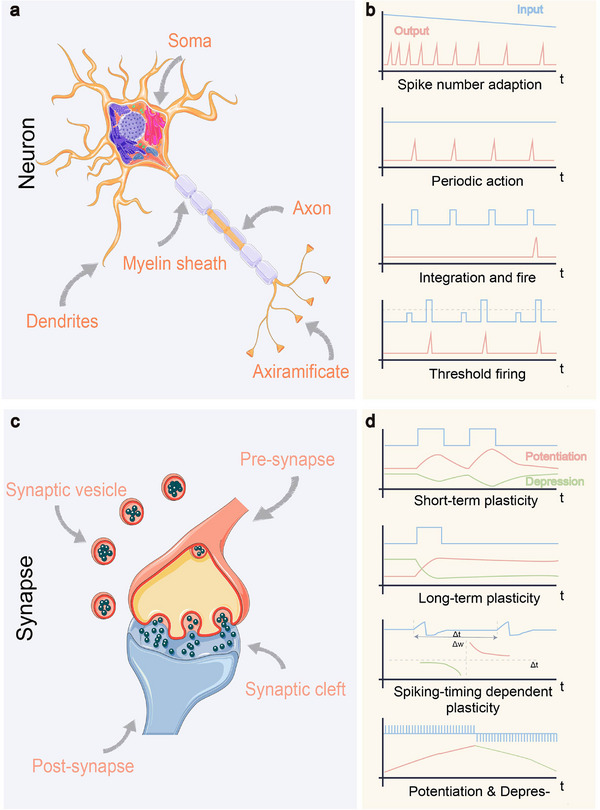
Biological and artificial neural systems. a) Schematic illustration of a biological neuron. b) Simulated curves of typical neuronal firing behaviors. c) Schematic illustration of a biological synapse. d) Simulated curves of typical synaptic plasticity behaviors.

Artificial neurons are neuromorphic functional units designed to emulate the membrane potential integration and threshold‐triggered firing behaviors of biological neurons (Figure 9a,b). Their defining characteristics include the spatiotemporal integration of multiple input signals, all‐or‐none spike generation governed by intrinsic threshold dynamics, pronounced nonlinear responses, and event‐driven operation that enables intrinsically energy‐efficient computation. In contrast to artificial synapses, which primarily mediate weight storage and synaptic plasticity, artificial neurons are responsible for nonlinear information integration and decision making, thereby serving as indispensable computational nodes within complete brain‐inspired computing architectures [152, 153, 154, 155].

In this device architecture, graphene serves as a continuous, low‐resistance two‐dimensional conductive layer, providing a stable electrode interface and uniform current injection pathway for the vertical MoS_2_, while avoiding interfacial damage that may be introduced by conventional metal electrodes. This configuration is therefore conducive to the reliable triggering of threshold‐switching behavior. Based on the volatile threshold‐switching characteristics exhibited by the v‐MoS_2_/graphene device, integrate‐and‐fire artificial neuron functionality was realized by incorporating a simple external RC circuit, including threshold‐triggered all‐or‐nothing spiking, refractory behavior, and firing‐frequency responses modulated by the amplitude of input pulses. Further studies revealed that the statistical distribution of threshold voltages during cycling originates from grain‐boundary‐related ion migration processes in the polycrystalline v‐MoS_2_ structure, thereby endowing the artificial neuron with a certain degree of stochastic firing behavior [156]. Compared with continuous two‐dimensional graphene films, GQDs exhibit pronounced quantum confinement effects arising from their size limitation, abundant and tunable edge/surface states, and markedly enhanced charge trapping and release capabilities [157]. In neuron devices, GQDs can effectively regulate membrane potential accumulation and firing thresholds by introducing discrete energy levels and dynamic trap states, thereby enabling more flexible threshold modulation, programmable stochasticity, and time‐dependent responses to input stimuli at the device level. In addition, the excellent solution processability of GQDs and their high material compatibility with a wide range of inorganic and organic semiconductor systems endow them with significant potential for integration into multi‐terminal and heterojunction‐type artificial neuron devices. Collectively, these attributes make it possible for graphene‐based QD devices to achieve synergistic control over energy consumption, functional density, and firing dynamics, thereby paving the way for low‐power, highly integrated neuromorphic devices that more closely emulate biological neurons in terms of temporal response and nonlinear characteristics.

### 5.4 Artificial Synaptic Devices

Biological synapses are the interfacial junctions through which neurons transmit signals, serving as the fundamental functional units that enable communication, learning, and memory in the nervous system. The degree of association between adjacent neurons is determined by the strength of their synaptic connections. Artificial synaptic devices mimic the signal‐transmission and plasticity mechanisms of biological synapses by modulating their physical parameters (e.g., electrical conductance), thereby enabling functions such as information storage, transmission, and learning (Figure 9c,d) [158, 159].

#### 5.4.1 Electronic Synaptic Devices

Electronic synapses represent the most mature class of artificial synaptic technologies, employing semiconductor materials to emulate the functions of biological synapses through electrical signals [160]. GQDs possess a high specific surface area and abundant edge‐active sites, enabling the formation of uniformly distributed charge‐trapping centers that effectively reduce parameter variability in memristive devices, thereby allowing precise emulation of synaptic weight modulation. Their quantum‐confinement and edge effects permit conductance tuning through control of size and surface functional groups, enabling multilevel resistive states that accommodate the requirements of synaptic plasticity. Moreover, GQDs exhibit high sensitivity to weak electrical stimuli due to their strong surface charge‐trapping capability, significantly lowering device operating voltage and power consumption, making them highly suitable for low‐power neuromorphic computing systems.

**FIGURE 10:**
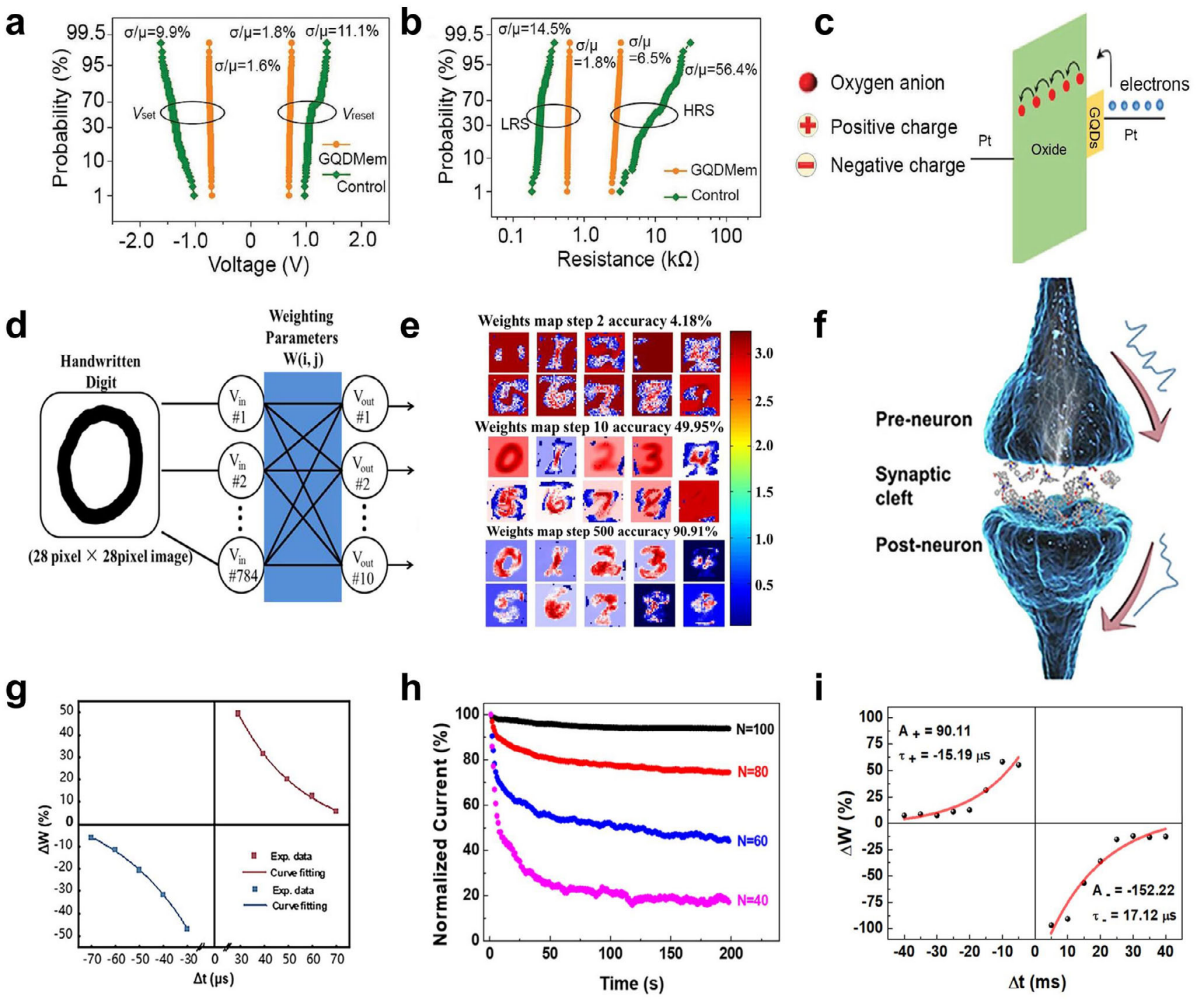
Artificial synapses based on graphene‐based QDs. a) Distribution of set and reset voltages for GQDMem and control memristors over 100 cycles. b) Distribution of HRS and LRS values for GQDMem and control memristors over 100 cycles. c) Energy band diagram of GQDMem under applied bias. Reproduced with permission [161]. Copyright 2017, John Wiley and Sons. d) The single‐layer architecture of ANN systems. e) The relationship between different training times and accuracy. Reproduced with permission [162]. Copyright 2020, John Wiley and Sons. f) Schematic of biological synapse structure. g) Relationship between synaptic weight and the time difference between pre‐ and post‐synaptic pulses, confirming that the device exhibits a standard STDP learning rule. Reproduced with permission [163]. Copyright 2025, The Royal Society of Chemistry. h) Normalized current after varying numbers of pulses, illustrating the transition from STM to LTM. i) Dependence of the synaptic weight on the spike time in STDP. Reproduced with permission [164]. Copyright 2024, Elsevier.

By exploiting the ability of GQDs to modulate the device band structure and enhance tunneling currents, Zhao et al. incorporated GQDs into memristive devices and developed GQD‐based memristors exhibiting highly repeatable analog resistive states. The device achieved a ∼40% reduction in switching voltage, an 84% narrowing of voltage variability, and an ∼85% reduction in analog‐state fluctuations, enabling highly controllable and reproducible analog resistance states (Figure 10a–c). Such devices are promising candidates for high‐precision and energy‐efficient artificial synapses in artificial neural networks [161]. To further improve the uniformity of resistive switching parameters, Yan et al. introduced GOQDs into the HfO_2_ dielectric layer as a stability‐enhancement layer and fabricated an Ag/HfO_2_/GOQD/Pt memristor. The device successfully emulated synaptic learning functions through its electrical characteristics, and achieved a recognition accuracy of 90.91% on the MNIST handwritten‐digit task after 500 training epochs (Figure 10d,e), outperforming some conventional RRAM‐based implementations [162]. In another study on GQD‐based memristors utilizing donor–acceptor (D–A) structures covalently modified with metalloporphyrins, Zhang et al. employed a covalent functionalization strategy to couple zinc porphyrin (ZnTPP) with GQDs, forming a D‐A hybrid material. Using this material, they fabricated a dual‐mode memristor with an Al/ZnTPP‐g‐GQDs:PVP/ITO structure. The device exhibited digital switching at high voltages (ON/OFF ratio > 10^2^, endurance of 200 cycles) and highly controllable analog behavior under low voltages or pulsed stimuli, achieving 50 distinct non‐volatile conductance states and emulating various synaptic learning and forgetting behaviors (Figure 10f,g). This dual‐mode capability, combining high‐precision analog modulation with stable digital storage, was successfully applied to a convolutional neural network, achieving a classification accuracy of 94.66% for five animal categories. The results highlight the strong potential of these devices in energy‐efficient neuromorphic computing and artificial‐intelligence hardware [163].

To further enhance the stability and electrical performance of memristive devices, Kim et al. incorporated chlorine‐functionalized GQDs (fGQDs) into a poly(methyl methacrylate) (PMMA) matrix to construct a sandwich‐structured Al/PMMA–fGQD/ITO memristive synaptic device. The results showed that at an fGQD doping concentration of 5 wt%, the device exhibited optimal performance with an ON/OFF ratio of 10^3^, and successfully reproduced a wide range of biological synaptic behaviors, including LTP/long‐term depression (LTD), short‐term/long‐term memory transitions, PPF, and spike‐timing‐dependent plasticity (STDP) (Figure 10h,i). Based on the device's conductance response, a single‐layer neural network constructed using this synapse achieved ∼90% recognition accuracy on the MNIST handwritten dataset after 50 training epochs, demonstrating its application potential in tasks such as image recognition and pattern classification [164]. Learning and forgetting are hallmark features of the brain, and faithfully emulating such functions is essential for neuromorphic devices to achieve brain‐like intelligence. This requires that neuromorphic devices not only reproduce the fundamental behaviors of biological synapses but also accurately model their dynamic modulation and higher‐order learning rules. Only through such deep and comprehensive biomimicry can these devices inherently achieve a dynamic balance between stability and plasticity, thereby granting artificial systems key capabilities such as continual learning, adaptive regulation, and resistance to catastrophic forgetting [165, 166, 167]. To address catastrophic forgetting in deep neural networks, Zhang et al. used a GQD‐based interfacial engineering strategy to fabricate a two‐terminal artificial synapse with enhanced metaplasticity. This device enabled programmable and linear weight updates with high repeatability, where the degree of modulation depended on the synapse's prior weight history. In continual learning tasks, neural networks employing this device achieved an accuracy of 97% on the fourth MNIST task while retaining above 94% accuracy on previous tasks, effectively mitigating catastrophic forgetting. Through GQD‐mediated asymmetric conductive behavior, the device replicated higher‐order plasticity dependent on historical activity in biological synapses, embedding metaplasticity mechanisms directly into deep‐learning architectures and narrowing the functional gap between artificial and biological neural systems [168].

#### 5.4.2 Optoelectronic Synaptic Devices

In the human visual cortex, neural networks composed of interconnected synapses excel at parallel processing tasks such as pattern recognition, with synaptic plasticity playing a central role. Studies have shown that in normal primates, over 80% of sensory information is acquired through vision. Therefore, developing optoelectronic synaptic devices that integrate light sensing, memory, and signal processing functions is crucial for constructing efficient, low‐power artificial visual neural networks [169, 170, 171, 172]. QDs, with tunable bandgaps, high luminescence efficiency, and size‐dependent optical absorption properties, have been widely explored in optoelectronic and neuromorphic devices. In photonic synaptic transistors, these nanomaterials enable spectrally selective responses and light‐controlled conductance modulation, making them suitable for artificial visual perception systems [31]. However, conventional QDs face challenges such as heavy‐metal toxicity, poor environmental stability, and uneven film interfaces. GQDs, by contrast, combine excellent chemical and photostability, broad ultraviolet absorption, low toxicity, and good solution‐processable compatibility, making them a more promising alternative material [173].

**FIGURE 11:**
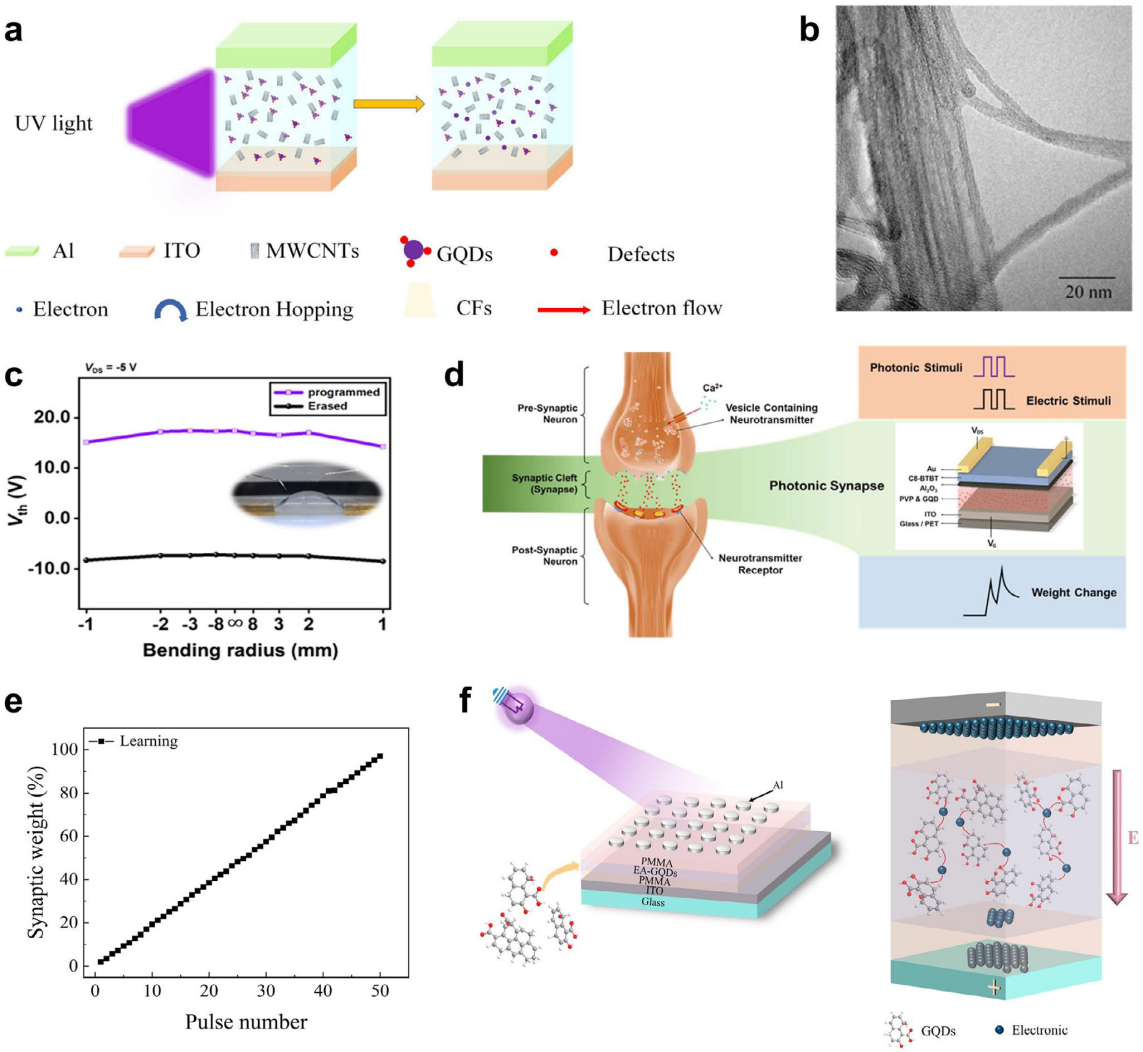
Optoelectronic synaptic devices based on graphene‐based QDs. a) The effect of UV on the dielectric film. b) TEM images of MWCNTs at high resolution. Reproduced with permission [174]. Copyright 2021, Multidisciplinary Digital Publishing Institute. c) Comparison of Vth variation in the flexible device under different bending radius in both tensile and compressive modes during photo‐programming (365 nm, 0.12 mWcm^−2^, and 10 s width) and electrical‐erasing (V_GS_ = −90 V, and 1 s width) operations. d) Schematic illustration of functional and structural comparisons of biological synapse with photonic synapse transistor in this work. Reproduced with permission [125]. Copyright 2025, American Chemical Society Publications. e) Learning behavior. f) Conduction mechanism of the Al/PMMA/EA–GQDs/PMMA/ITO device under UV illumination. Reproduced with permission [175]. Copyright 2023, Multidisciplinary Digital Publishing Institute.

Exploiting the photo‐tunable charge‐trapping capability of GQDs, Wen et al. fabricated a dual‐modulation memristor with an Al/MWCNT:GQD/ITO structure using spin‐coating and vacuum deposition, where a composite of multiwalled carbon nanotubes (MWCNTs) and GQDs served as the dielectric layer (Figure 11b). The device enables dual‐mode control of the switching ratio: increasing the GQD content (from 1:0.125 to 1:0.5) raises the ON/OFF ratio from 11 to 3.3 × 10^3^, while UV illumination (0 to 30 min) reduces the ON/OFF ratio from 4.58 × 10^3^ to 10.77 (Figure 11a). In addition, the device demonstrated over 100 stable write/erase cycles, data retention exceeding 10^4^ s, and excellent endurance under 10^5^ read pulses. These characteristics highlight its broad potential for applications in multilevel data storage, tunable logic memories, artificial neural networks, and low‐power artificial intelligence hardware [174]. Leveraging the tunable bandgap and strong photoluminescence of GQDs, Park et al. fabricated a flexible ultraviolet‐responsive optoelectronic synaptic transistor using functionalized GQDs (HBC‐PF6 GQDs), exploiting their light absorption and charge‐trapping properties to integrate photodetection, memory, and signal processing. The device successfully emulated multiple synaptic behaviors at ultralow energy consumption (1.2 fJ per event), including excitatory postsynaptic current (EPSC), PPF, short‐term to long‐term plasticity transitions, and reversible LTP/LTD (Figure 11d). It maintained stable operation under extreme bending radii (±1 mm) (Figure 11c) and achieved a 91% recognition accuracy on handwritten digits using an artificial neural network. Through its flexible substrate, low‐power neuromorphic design, and stable UV response, this device provides a promising strategy for wearable ultraviolet neuromorphic visual systems [125]. Owing to their excellent biocompatibility and low cytotoxicity, GQDs possess inherent advantages as dopants for biomaterials. Wen et al. fabricated an Al/PMMA/EA–GQDs/PMMA/ITO optoelectronic dual‐modulation bio‐memristor using egg albumin (EA) as the dielectric layer, GQDs as the photosensitive dopant, and PMMA as the top and bottom insulating layers via solution‐based spin coating (Figure 11f). The device exhibited stable resistive switching behavior (ON/OFF current ratio ∼10^4^) and, under electrical stimulation and UV illumination, successfully emulated nine synaptic functions, including EPSC, PPF, STP/LTP, STDP, and Pavlovian associative memory (Figure 11e). With its excellent stability, plasticity, and biocompatibility, this device provides a new materials and device platform for optoelectronic neuromorphic computing and brain‐inspired intelligent systems [175].

#### 5.4.3 Flexible Biocompatible Neuromorphic Devices

Synapses in biological neural systems possess inherent flexibility and deformability, enabling them to adapt to continuous mechanical changes in tissues. To approach the energy efficiency of biological systems, artificial neuromorphic devices must also exhibit corresponding bendable and stretchable physical properties, meeting the requirements of bioinspired soft electronics and wearable intelligent devices [176, 177, 178, 179, 180]. GQDs, with their excellent flexibility, mechanical stability, low processing temperature, and abundant functional surface structures, make them ideal materials for constructing flexible neuromorphic devices.

**FIGURE 12:**
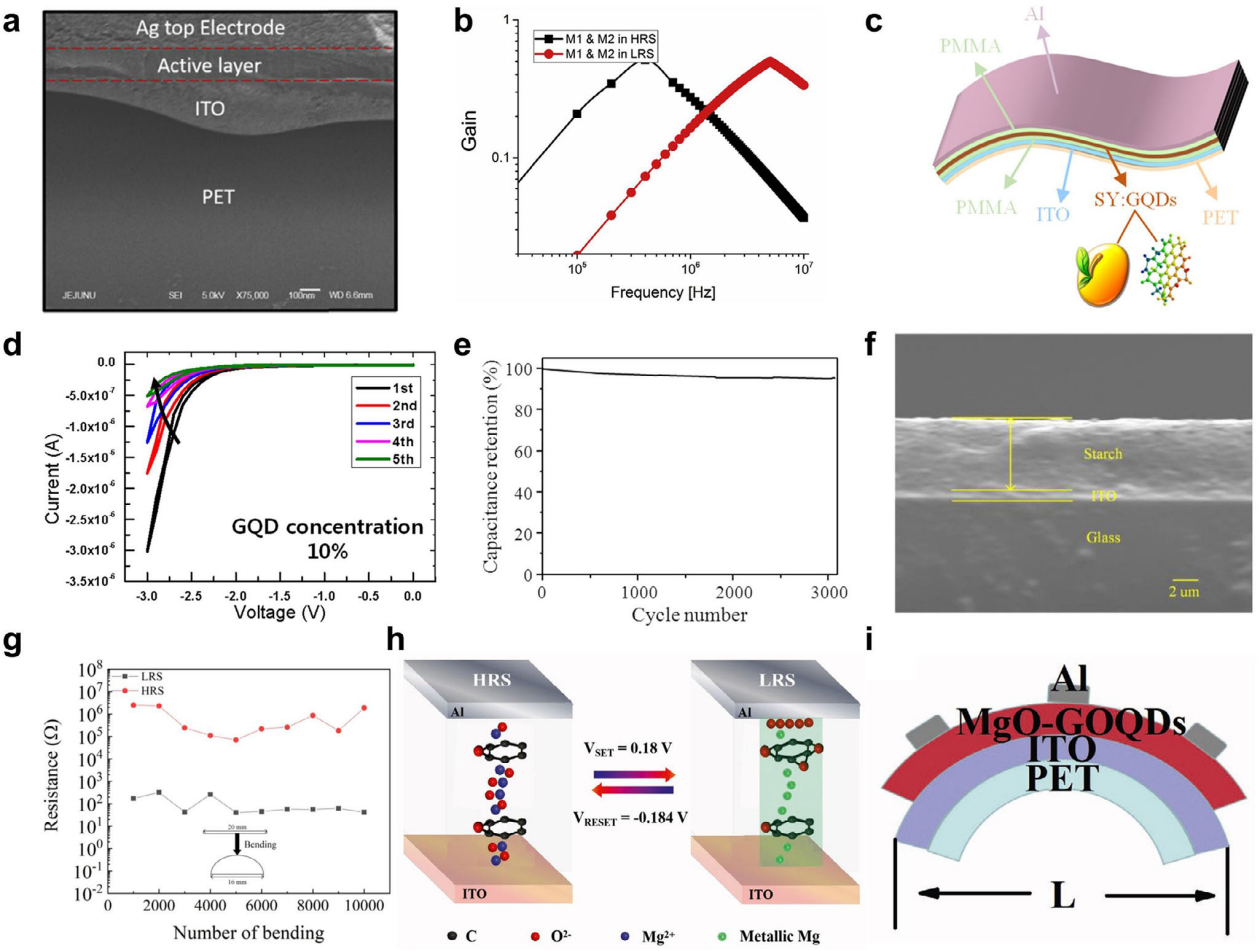
Flexible biocompatible neuromorphic devices based on graphene‐based QDs. a) Cross‐sectional image of the memristor, showing the three‐layer structure of Ag/active layer (GQDs/PVP)/ITO. b) Band‐pass filter response with M1 and M2 in the HRS and with M1 and M2 in the LRS. Reproduced with permission [181]. Copyright 2017, Elsevier. c) Structure diagram of the Al/PMMA/SY:GQDs/PMMA/ITO/PET. Reproduced with permission [182]. Copyright 2023, The Royal Society of Chemistry. d) *I–V* curves of the ITO/CEA:GQD/Al devices during five voltage sweeps for GQD concentrations of 10%. Reproduced with permission [183]. Copyright 2020, Springer Nature. e) Capacitance retention after 3000 cycles in 1 M KOH. Reproduced with permission [185]. Copyright 2016, Springer Nature. f) SEM images of starch film cross‐section. g) R_LRS_ and R_HRS_ after 10^4^ device bends. Reproduced with permission [186]. Copyright 2023, Multidisciplinary Digital Publishing Institute. h) Illustration depicting the resistive switching mechanism within the MgO‐GOQD memristive device. i) Schematic representation of the flexible Al/MgO‐GOQD/ITO device fabricated on a PET substrate. Reproduced with permission [187]. Copyright 2021, John Wiley and Sons.

To address the issue of LRS and HRS drift over endurance cycles in memristors, Lee et al. employed electrohydrodynamic printing to fabricate a passive MC filter on a flexible PET substrate, integrating a graphene quantum dot/PVP memristor with a graphene/PVP capacitor (Figure 12a). The device can modulate the filter's cutoff frequency and bandwidth by switching between HRS and LRS, achieving a tunable frequency range from several hundred kHz to a few MHz (Figure 12b). Moreover, it demonstrated stable electrical performance under mechanical bending tests with a minimum bending diameter of 8 mm [181]. Flexible bio‐memristors with low cost, biodegradability, and solubility hold great potential for future medical and bioinspired applications. Wen et al. developed a flexible bio‐memristor using a soybean (SY)–GQD composite as the active layer, sandwiched between PMMA insulating layers (Figure 12c). The device exhibited an exceptionally high ON/OFF ratio of 9.17 × 10^6^, low‐voltage operation below –0.9 V, and excellent bending durability, maintaining stable performance after 10^4^ bending cycles. By modulating the Schottky barrier between the electrodes and PMMA, the device enabled controllable resistive switching, and individual units could perform “AND” and “OR” logic operations, realizing in‐memory computing. Fabricated from natural soybean (SY) materials, the device is degradable, non‐toxic, and biocompatible, making it suitable for skin‐contact or implantable applications and reducing the biorejection risks associated with conventional electronic materials [182]. Leveraging the remarkable charge‐storage capability of GQD‐based hybrid nanocomposites, Kim et al. used spin coating to fabricate a bio‐synaptic device with an ITO/CEA:GQD/Al structure, employing a nanocomposite of chicken egg albumin (CEA) and GQDs as the active layer. At an optimized GQD concentration of 10%, the device exhibited a characteristic clockwise shrinking hysteresis, stably emulating LTD in biological synapses, with a retention time exceeding 104 s and excellent cycling stability (Figure 12d). This bio‐synaptic device is suitable for neuromorphic computing and low‐power brain‐inspired hardware systems, providing a feasible approach for constructing biocompatible artificial synapses [183]. According to previous studies, most synthesized GQDs are hydrophilic, which to some extent limits their applications in organic electronics and energy storage devices [184]. Chen et al. employed a one‐pot hydrothermal method to simultaneously exfoliate and dissociate graphite sheets, synthesizing hydrophilic nitrogen‐doped GQDs (IN‐GQDs) and hydrophobic nitrogen‐doped GQDs (ON‐GQDs). These materials were then used to fabricate high‐performance supercapacitors and bio‐memristors, respectively. The flexible paper electrode composed of ON‐GQDs and single‐walled carbon nanotubes achieved an impressive areal capacitance of 114 mF/cm^2^, over 250% higher than the best reported values, and retained 95% of its capacitance after 3000 cycles (Figure 12e). Meanwhile, the bio‐memristor fabricated from IN‐GQDs and proteins exhibited an ON/OFF ratio exceeding 10^4^, stable switching over 250 cycles, and retention of resistive states for more than 104 s. Additionally, IN‐GQDs demonstrated a high quantum yield of 34%, excellent stability for cellular imaging, and negligible cytotoxicity. This study provides a new materials platform and feasible approach for carbon‐based supercapacitors, non‐volatile bio‐storage devices, and bioimaging applications [185].

Although bio‐memory devices offer numerous advantages, their stability and reliability generally require improvement. Wen et al. employed a strategy of incorporating GQDs into a starch‐based dielectric layer, with PMMA modulation layers introduced at both interfaces, to fabricate an Al/starch:GQDs/ITO memristor (Figure 12f). The device exhibited an ON/OFF current ratio exceeding 10^5^, with the reset voltage significantly reduced to –0.75 V. Individual memory units could endure over 120 repeated switching cycles, and flexible devices on PET substrates maintained stable bipolar resistive switching even after 10,000 bending cycles (Figure 12g) [186]. To further enhance flexible memory performance, Seo's research team utilized reduced graphene oxide quantum dots (rGOQDs) combined with a self‐assembled monolayer of Au nanoparticles as a charge‐trapping mediation layer to successfully fabricate memory‐capable capacitors and transistor devices on flexible substrates. This approach enabled high‐performance non‐volatile storage using an all‐solution, low‐temperature process. The devices exhibited pronounced non‐bipolar transport and tunable memory characteristics: the capacitors showed a flat‐band voltage shift of 3.74 V under ±10 V scans, while the transistors displayed a threshold voltage shift of 7.84 V with quasi‐saturation output behavior, an ON/OFF ratio of 10, and a mobility of 5.005 cm^2^/V·s. The devices maintained electrical stability under bending; during a 1 cm bending radius test on a flexible PET substrate, the rGOQD‐based transistor showed no change in threshold voltage shift [120]. Beyond memory functionality, quantum dot nanocomposites also demonstrate significant potential in neuromorphic computing. Kim et al. doped GOQDs into a PVP matrix to construct a synaptic device based on the PVP/GOQD nanocomposite. The device accurately emulated multiple synaptic functions, including LTP/LTD, PPF, post‐tetanic potentiation (PTP), and transitions from short‐term to long‐term memory. It maintained stable synaptic behavior at bending radii of 5 mm and 10 mm, with no significant performance degradation after 3000 bending cycles. When applied to MNIST learning and inference tasks, the device achieved a high accuracy of 89.39%. Structurally, the device employs a PVP/GOQD nanocomposite thin film as the functional layer, simulating synaptic weight updates through controlled formation and rupture of silver conductive filaments. This work offers a promising approach for advancing flexible neuromorphic computing and brain‐inspired chips, providing a feasible pathway for achieving high‐reliability artificial synapses and fully flexible intelligent systems [112]. Furthermore, through material hybridization and structural engineering, it is possible to enhance the electrical and synaptic performance of devices while maintaining flexibility. Zhao et al. prepared a magnesium oxide–graphene oxide quantum dot (MgO‐GOQD) composite film via a solution‐based method and fabricated a memristive artificial synapse based on this material (Figure 12i). The study demonstrated that GOQDs can modulate the resistive switching process by enhancing local electric fields and exhibiting redox behavior under an applied field (Figure 12h). Moreover, the device retained stable resistive switching characteristics and synaptic functions including PPF, STP/LTP transitions, and STDP after 500 bending cycles. This work highlights the significant potential of solution‐processed MgO‐GOQD hybrid films for flexible artificial neural networks [187].

## 6 Conclusion and Outlook

**FIGURE 13:**
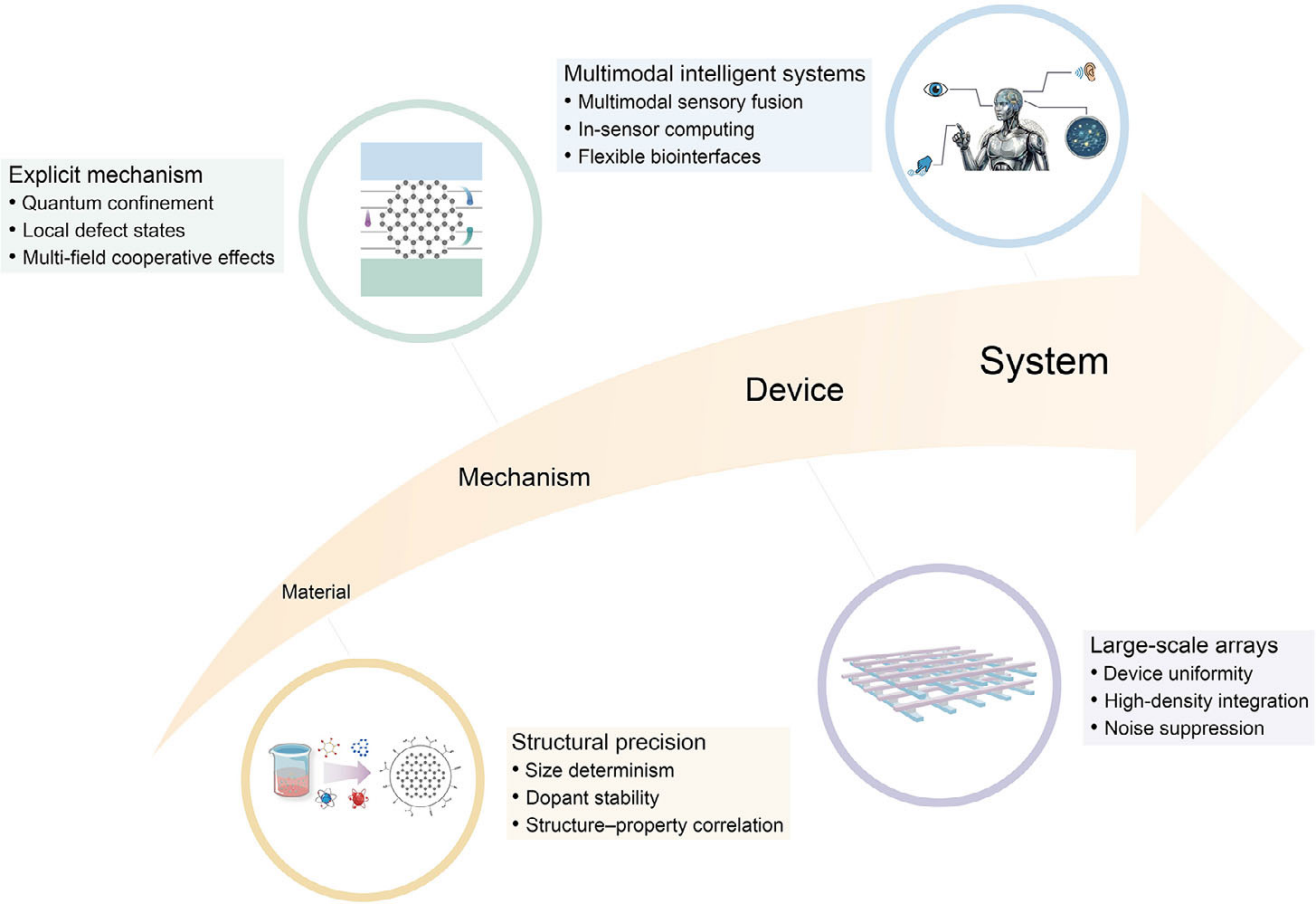
Development directions of graphene‐based QDs in neuromorphic devices.

In summary, this review systematically summarizes the controllable synthesis strategies of graphene‐based QDs and their tunable physicochemical properties, focusing on the modulation mechanisms of graphene‐based QDs in various neuromorphic device architectures, as well as recent progress of graphene‐based QDs in artificial synapses, optoelectronic neuromorphic devices, flexible systems, and bio‐integrated computing platforms. Benefiting from quantum confinement effects, abundant surface states, and excellent interfacial compatibility, graphene‐based QDs exhibit unique advantages in enabling low‐power operation, multimodal responsiveness, and flexible adaptability. These features provide important guidance for the design of next‐generation neuromorphic hardware. However, despite considerable progress, neuromorphic devices based on graphene‐based QDs still face key challenges in scaling up and practical deployment, requiring further systematic breakthroughs (Figure 13).

(1) Enhancing structural precision of graphene‐based QDs

Although existing synthesis methods can produce usable quantum dots, significant variations remain in size distribution, defect density, heteroatom doping stability, and surface functional group control. These structural differences directly affect charge‐trapping behavior, ion migration dynamics, and photocarrier processes in neuromorphic devices. Given the critical role of defect states and surface groups in synaptic plasticity, a deeper understanding of their formation mechanisms and interactions with device interfaces is urgently needed. Moreover, the stability of graphene‐based QDs under oxidation, humidity, and intense illumination must be improved, especially for flexible or wearable devices. Future studies should focus on green and efficient synthesis, quantitative energy‐level/defect engineering, and systematically elucidating the intrinsic relationship between structure and device performance.

(2) Elucidating multi‐scale coupling effects in devices based on graphene‐based QDs

Graphene‐based QDs can emulate synaptic behavior through charge trapping, ion rearrangement, interfacial dipole modulation, or photo‐induced processes; however, the coupling between these mechanisms remains unclear. Current devices also commonly exhibit nonlinear weight updates, significant device‐to‐device variability, and insufficient cycling stability, which are far from meeting the consistency and reliability requirements of large‐scale neuromorphic computing. Understanding how quantum confinement, local defect states, and multi‐field cooperative effects jointly influence synaptic dynamics is of vital importance. As neuromorphic computing increasingly targets biologically relevant learning mechanisms, such as metaplasticity and homeostatic regulation, there is an urgent need to construct device systems based on graphene‐based QDs capable of supporting complex learning rules and state evolution.

(3) Realizing high‐consistency GQD neuromorphic hardware for large‐scale arrays

Most studies to date focus on single devices or small arrays. Scaling up introduces amplified issues such as parasitic interference, signal attenuation, and thermal drift. Additionally, the compatibility of materials based on graphene‐based QDs with CMOS back‐end processes requires further verification. While novel structures, such as GQD/2D semiconductor heterojunctions and GQD‐doped polymer electrolytes, may enhance linearity, reduce power consumption, and improve uniformity, the lack of standardized testing makes direct comparisons difficult. For scalable deployment, strategies for high‐density integration, reliable interconnects, and noise suppression must be developed to enable practical implementation of GQD neuromorphic arrays.

(4) Toward multimodal intelligent systems based on graphene‐based QDs

Research on integrating sensing, memory, and processing functions within a single system – such as artificial vision, implantable neural interfaces, and smart wearable devices – remains limited. Moreover, device energy consumption, long‐term biocompatibility, and system‐level online learning capabilities under real‐world conditions are still insufficiently evaluated. Given the inherent photoresponse and mechanical flexibility of graphene‐based QDs, future work can further develop multimodal sensory fusion, in‐memory computing architectures, and skin‐inspired intelligent electronics to better emulate biological perception. Material innovation, device structure optimization, co‐design of system architectures, and machine‐learning‐driven operational strategies will collectively drive GQD neuromorphic systems toward high‐density integration, low‐power operation, and functional diversity (Table 1).

**TABLE 1:** Graphene‐based QDs for Non‐Volatile Memory and Artificial Synaptic Device.

Structure	Endurance	Retention time	ON/OFF Ratio	Synaptic function	Energy Consumption	Application	Refs.
Ag/GQDs:GO/ITO	> 500 cycles	—	> 10^3^	—	—	memory device	[188]
ITO/GO:GQDs/Ni	> 100 cycles	10^4^ s	10^3^:10^2^:1	—	—	memory device	[142]
Al/PEDOT‐PSS:GOQDs/ITO	≈ 200 cycles	10^4^ s	10^4^	—	—	memory device	[107]
Au/TPAPAM‐GOQDs/ITO	> 10^6^ cycles	10^4^ s	3000:60:1	—	17.65 pJ/pulse	memory device	[189]
Ag/GQDs/TiOx/FTO	> 300 cycles	—	—	LTP/LTD	—	memory device	[190]
Pd/Al_2_O_3_/HfO_2_/GOQDs/HfO_2_/SiO_2_/Si	> 10^5^ cycles	1.2 × 10^4^ s	—	—	—	memory device	[141]
crossbar Ag/PFG/PVA/Ag	> 200 cycles	—	10^4^–10^5^	—	—	memory device	[146]
Ag/GQDs/PVP/Ag	> 500 cycles	210 days	∼14	—	—	memory device	[147]
Ag/ZHO/GOQDs/ZHO/Pt	> 10^6^ cycles	10^4^ s	∼10^4^	—	0.0585 pJ/pulse	memory device	[148]
Al/N‐GQDs/PVA nanocomposite thin film/Ag	> 50 cycles	10^4^ s	∼10^2^	—	—	memory device	[151]
Al_2_O_3_/GQD/Al_2_O_3_	> 100 days	—	—	STD/LTP/PPF	< 160 pJ/Spike	synaptic device	[191]
Ag/PI:GQDs/ITO	> 100 cycles	—	—	STD/PPF/LTP	—	synaptic device	[108]
Ag/Zr_0.5_Hf_0.5_O_2_ZHO/GOQDs/ZHO/Pt	> 100 cycles	—	—	STP/STDP/PPF	—	synaptic device	[192]
Ag/GO/BP·GOQD/GO/ITO	> 3 months	—	—	PPF/STDP/STDP/LTM	12.4 pJ/pulse	synaptic device	[109]
Ag/ZHO/GOQDs/ZHO/Ag	> 100 cycles	—	—	STDP/PPF/STP/LTP	8 pJ/pulse	synaptic device	[113]
GQDs/Au/graphene/ITO/PI	> 200 cycles	—	—	LTP/LTD	—	synaptic device	[44]
Ag/N‐GOQDs/Pt	12 000 pulse cycles	—	∼10^7^	STP/PPF/LTD/STDP	—	synaptic device	[114]
Ag/N‐GOQDs/Pt	> 5 × 10^3^ AC pulse cycles	—	∼10^6^	EPSC/STP/LTP	—	synaptic device	[127]
Al/ZnTPP‐g‐GQDs:PVP/ITO	> 200 cycles	10^4^ s	∼10^3^	STP/LTP/STDP/SRDP/PPF/PTP	—	synaptic device	[163]
Ag/HfO_2_/GOQD/Pt	> 200 cycles	—	∼10^6^	STP/LTP/EPSC/PPF	—	synaptic device	[162]

In conclusion, although neuromorphic devices based on graphene‐based QDs have advanced rapidly, their transition from laboratory prototypes to scalable manufacturing and practical deployment still faces multidimensional bottlenecks, including material controllability, mechanistic interpretability, device uniformity, and system‐level synergy. In the future, as precise quantum‐structure control, heterointerface engineering, and multi‐physical coupling design mature, together with evolving hardware‐algorithm co‐design paradigms for brain‐inspired computing, graphene‐based QDs are expected to play a pivotal role in constructing next‐generation, ultra‐low‐power, high‐density, online‐learning‐capable neuromorphic hardware, providing breakthrough materials foundations and technological pathways for post‐Moore intelligent computing systems.

## Conflicts of Interest

The authors declare no conflict of interest.

## References

[1] Y. LeCun , Y. Bengio , and G. Hinton , “Deep Learning,” Nature 521 (2015): 436–444, 10.1038/nature14539. 26017442

[2] Y.‐J. Chen and C.‐F. Chien , “An Empirical Study of Demand Forecasting of Non‐volatile Memory for Smart Production of Semiconductor Manufacturing,” International Journal of Production Research 56 (2018): 4629–4643, 10.1080/00207543.2017.1421783.

[3] D. Kim and J.‐S. Lee , “Liquid‐Based Memory Devices for Next‐Generation Computing,” ACS Applied Electronic Materials 5 (2023): 664–673, 10.1021/acsaelm.2c01636.

[4] Y. Liu , M. Lian , W. Chen , and H. Chen , “Recent Advances in Fabrication and Functions of Neuromorphic System Based on Organic Field Effect Transistor,” International Journal of Extreme Manufacturing 6 (2024): 022008, 10.1088/2631-7990/ad1e25.

[5] H. S. P. Wong and S. Salahuddin , “Memory Leads the Way to Better Computing,” Nature Nanotechnology 10 (2015): 191–194, 10.1038/nnano.2015.29. 25740127

[6] I.‐J. Kim , M.‐K. Kim , and J.‐S. Lee , “Highly‐scaled and Fully‐integrated 3‐dimensional Ferroelectric Transistor Array for Hardware Implementation of Neural Networks,” Nature Communications 14 (2023): 504, 10.1038/s41467-023-36270-0. PMC9889761 36720868

[7] C. Hwang , “Prospective of Semiconductor Memory Devices: from Memory System to Materials,” Advanced Electronic Materials 1 (2015): 1400056, 10.1002/aelm.201400056.

[8] P. Sun , R. Li , H. Meng , T. Sun , S. Gao , and Y. Li , “ZnO‐SnO_2_/WO_3‐x_ Heterojunction Artificial Synapse for Realization and Integration of Multiple Biological Cognitive Functions,” International Journal of Extreme Manufacturing 7 (2025): 055505, 10.1088/2631-7990/addf1e.

[9] N. Sui , K. Kang , M. Li , et al., “Fabrication of Carbon Nanotube Neuromorphic Thin Film Transistor Arrays and Their Applications for Flexible Olfactory‐visual Multisensory Synergy Recognition,” International Journal of Extreme Manufacturing 7 (2025): 015503, 10.1088/2631-7990/ad8737.

[10] Z. Huo , Q. Sun , J. Yu , et al., “Neuromorphic Devices Assisted by Machine Learning Algorithms,” International Journal of Extreme Manufacturing 7 (2025): 042007, 10.1088/2631-7990/adba1e.

[11] X. Lin , Z. Feng , Y. Xiong , et al., “Piezotronic Neuromorphic Devices: Principle, Manufacture, and Applications,” International Journal of Extreme Manufacturing 6 (2024): 032011, 10.1088/2631-7990/ad339b.

[12] B. Sengupta , M. Stemmler , S. B. Laughlin , and J. E. Niven , “Action Potential Energy Efficiency Varies among Neuron Types in Vertebrates and Invertebrates,” PLOS Computational Biology 6 (2010): 1000840, 10.1371/journal.pcbi.1000840. PMC2895638 20617202

[13] C. Mead , “Neuromorphic Electronic Systems,” Proceedings of the IEEE 78 (1990): 1629–1636, 10.1109/5.58356.

[14] R. Yu , L. He , C. Gao , et al., “Programmable Ferroelectric Bionic Vision Hardware with Selective Attention for High‐precision Image Classification,” Nature Communications 13 (2022): 7019, 10.1038/s41467-022-34565-2. PMC9669032 36384983

[15] S. Nam , D. Kang , J.‐W. Jo , D.‐W. Kang , S. K. Park , and Y.‐H. Kim , “Recent Progress of Neuromorphic Sensory and Optoelectronic Systems,” International Journal of Extreme Manufacturing 7 (2025): 042006, 10.1088/2631-7990/adbb33.

[16] P. A. Merolla , J. V. Arthur , R. Alvarez‐Icaza , et al., “A Million Spiking‐neuron Integrated Circuit with a Scalable Communication Network and Interface,” Science 345 (2014): 668–668, 10.1126/science.1254642. 25104385

[17] H. Zhong , Q.‐C. Sun , G. Li , et al., “High‐performance Synaptic Transistors for Neuromorphic Computing*,” Chinese Physics B 29 (2020): 040703, 10.1088/1674-1056/ab7806.

[18] M. A. Khan , S. Yim , S. Rehman , et al., “Two‐dimensional Materials Memory Devices with Floating Metal Gate for Neuromorphic Applications,” Materials Today Advances 20 (2023): 100438, 10.1016/j.mtadv.2023.100438.

[19] K. Xu and S. K. Fullerton‐Shirey , “Ionically Gated Transistors Based on Two‐dimensional Materials for Neuromorphic Computing,” 2D Materials 12 (2025): 023003, 10.1088/2053-1583/adb8c3.

[20] H. Xu , F. Sun , E. Li , et al., “Ferroelectric Perovskite/MoS_2_ Channel Heterojunctions for Wide‐Window Nonvolatile Memory and Neuromorphic Computing,” Advanced Materials 37 (2025): 2414339, 10.1002/adma.202414339. 39580680

[21] J. Bian , Z. Liu , Y. Tao , et al., “Advances in Memristor Based Artificial Neuron Fabrication‐materials, Models, and Applications,” International Journal of Extreme Manufacturing 6 (2024): 012002, 10.1088/2631-7990/acfcf1.

[22] C. Tian , Y. Cho , Y. Song , S. Park , I. Kim , and S.‐Y. Cho , “Integration of AI with Artificial Sensory Systems for Multidimensional Intelligent Augmentation,” International Journal of Extreme Manufacturing 7 (2025): 042002, 10.1088/2631-7990/adbd98.

[23] S. J. Kim , H.‐J. Lee , C.‐H. Lee , and H. W. Jang , “2D materials‐based 3D Integration for Neuromorphic Hardware,” npj 2D Materials and Applications 8 (2024): 70, 10.1038/s41699-024-00509-1.

[24] J.‐H. Kwag , S.‐H. Choi , D. Kim , et al., “Tailoring the Number of Lines for IGO‐channel 2T0C DRAM Comparable to Conventional 2‐line Operation 1T1C Structure for Highly Scaled Cell Volume,” International Journal of Extreme Manufacturing 7 (2025): 055503, 10.1088/2631-7990/add7a3.

[25] K. Nam , G. Y. Kim , D. Rhee , H. Park , D. Jariwala , and J. Kang , “Solution‐based Manufacturing of 2D Materials for Memristive Device Applications,” International Journal of Extreme Manufacturing 7 (2025): 052001, 10.1088/2631-7990/add634.

[26] Y. Lin , X. Chen , Q. Zhang , et al., “Nano Device Fabrication for in‐memory and in‐sensor Reservoir Computing,” International Journal of Extreme Manufacturing 7 (2025): 012002, 10.1088/2631-7990/ad88bb.

[27] Z. Huang , Y. Li , Y. Zhang , J. Chen , J. He , and J. Jiang , “2D multifunctional Devices: from Material Preparation to Device Fabrication and Neuromorphic Applications,” International Journal of Extreme Manufacturing 6 (2024): 032003, 10.1088/2631-7990/ad2e13.

[28] A. Taher , M. A. Rahman , R. Mia , et al., “Quantum Dot‐based Non‐volatile Memory: a Comprehensive Outlook,” RSC Advances 15 (2025): 14428–14462, 10.1039/d4ra08307e. 40330043 PMC12053827

[29] G. Liu , H. Liu , F. Fan , et al., “MoS_2_‐based Quantum Dot Artificial Synapses for Neuromorphic Computing,” Materials Today Physics 53 (2025): 101703, 10.1016/j.mtphys.2025.101703.

[30] D.‐H. Ngo and N. L. Nguyen , “Hydrogen‐driven Engineering of Electronic Properties in PbS Quantum Dots and Superlattices,” Journal of Physics and Chemistry of Solids 208 (2026): 113142, 10.1016/j.jpcs.2025.113142.

[31] G. K. Gupta , I.‐J. Kim , Y. Park , M.‐K. Kim , and J.‐S. Lee , “Inorganic Perovskite Quantum Dot‐Mediated Photonic Multimodal Synapse,” ACS Applied Materials & Interfaces 15 (2023): 18055–18064, 10.1021/acsami.2c23218. 37000192

[32] Y. Wang , C. Wang , X. Zhang , et al., “Dual‐functional Light Adaptation in Perovskite Quantum Dot Synaptic Devices for Smart Blue‐light Protection,” Science China Materials 69 (2026): 161–170, 10.1007/s40843-025-3582-9.

[33] H. Song , Y. Ru , J. Yu , et al., “Carbon Dots: from Fundamentals to Frontier Applications,” Science China Chemistry 69 (2026): 539–618, 10.1007/s11426-025-3195-y.

[34] A. Lakshmi‐Narayana , N. Attarzadeh , V. Shutthanandan , and C. V. Ramana , “High‐Performance NiCo_2_O_4_ /Graphene Quantum Dots for Asymmetric and Symmetric Supercapacitors with Enhanced Energy Efficiency,” Advanced Functional Materials 34 (2024): 2316379, 10.1002/adfm.202316379.

[35] M. Choppadandi , A. T. Guduru , P. Gondaliya , et al., “Structural Features Regulated Photoluminescence Intensity and Cell Internalization of Carbon and Graphene Quantum Dots for Bioimaging,” Materials Science and Engineering: C 129 (2021): 112366, 10.1016/j.msec.2021.112366. 34579885

[36] R. Riaz , M. Ali , I. A. Sahito , et al., “Self‐assembled Nitrogen‐doped Graphene Quantum Dots (N‐GQDs) over Graphene Sheets for Superb Electro‐photocatalytic Activity,” Applied Surface Science 480 (2019): 1035–1046, 10.1016/j.apsusc.2019.02.228.

[37] C.‐Y. Chang , S. Venkatesan , A. Herman , C.‐L. Wang , H. Teng , and Y.‐L. Lee , “Carbon Quantum Dots with High Quantum Yield Prepared by Heterogeneous Nucleation Processes,” Journal of Alloys and Compounds 938 (2023): 168654, 10.1016/j.jallcom.2022.168654.

[38] V. Kansara and M. Patel , “Insight into the Effect of Synthesis Parameters on the Properties of Graphene Quantum Dots for Theranostic Application,” Colloids and Surfaces A: Physicochemical and Engineering Aspects 684 (2024): 133097, 10.1016/j.colsurfa.2023.133097.

[39] M. Sharma , S. R. Kafle , A. Singh , A. Chakraborty , and B. S. Kim , “Carbon Quantum Dots for Efficient Hydrogen Production: a Critical Review,” Chemcatchem 16 (2024): 202400056, 10.1002/cctc.202400056.

[40] S.‐J. Jeon , T.‐W. Kang , J.‐M. Ju , et al., “Modulating the Photocatalytic Activity of Graphene Quantum Dots via Atomic Tailoring for Highly Enhanced Photocatalysis under Visible Light,” Advanced Functional Materials 26 (2016): 8211–8219, 10.1002/adfm.201603803.

[41] S. Mandal , S. Adhikari , M. Murmu , B.‐H. Kim , and D.‐H. Kim , “Graphene and Carbon Quantum Dots: Competing Carbons in Harmonized Photoelectrochemical Platforms,” Small 21 (2025): 05846, 10.1002/smll.202505846. PMC12508723 40820769

[42] P. Tian , L. Tang , K. S. Teng , and S. P. Lau , “Graphene Quantum Dots from Chemistry to Applications,” Materials Today Chemistry 10 (2018): 221–258, 10.1016/j.mtchem.2018.09.007.

[43] Y. Pei , J. Zhang , M. Guo , et al., “Low‐dimensional Optoelectronic Memristors: from Quantum Confinement to Neuromorphic Vision,” Materials Science and Engineering: R: Reports 167 (2026): 101115, 10.1016/j.mser.2025.101115.

[44] S. W. Hwang and D.‐K. Hong , “Flexible Memristive Devices Based on Graphene Quantum‐Dot Nanocomposites,” Computers, Materials & Continua 72 (2022): 3283–3297, 10.32604/cmc.2022.025931.

[45] V. Khademhosseini , D. Dideban , M. Ahmadi , and R. Ismail , “Current Analysis of Single Electron Transistor Based on Graphene Double Quantum Dots,” ECS Journal of Solid State Science and Technology 9 (2020): 021003, 10.1149/2162-8777/ab6980.

[46] M. Favaro , L. Ferrighi , G. Fazio , et al., “Single and Multiple Doping in Graphene Quantum Dots: Unraveling the Origin of Selectivity in the Oxygen Reduction Reaction,” ACS Catalysis 5 (2015): 129–144, 10.1021/cs501211h.

[47] P. C. Ricardo , G. Nicolodelli , and M. C. Fredel , “Tunable Photoluminescence of Graphene Quantum Dots Synthesized via Laser Ablation in Ethanol,” Journal of Luminescence 281 (2025): 121186, 10.1016/j.jlumin.2025.121186.

[48] P. Kadyan , M. Kumar , A. Tufail , A. Ragusa , S. K. Kataria , and A. Dubey , “Microwave‐assisted Green Synthesis of Fluorescent Graphene Quantum Dots (GQDs) Using Azadirachta Indica Leaves: Enhanced Synergistic Action of Antioxidant and Antimicrobial Effects and Unveiling Computational Insights,” Materials Advances 6 (2025): 805–826, 10.1039/D4MA00843J.

[49] D. Pan , J. Zhang , Z. Li , and M. Wu , “Hydrothermal Route for Cutting Graphene Sheets into Blue‐Luminescent Graphene Quantum Dots,” Advanced Materials 22 (2010): 734–738, 10.1002/adma.200902825. 20217780

[50] H. Kalita , V. S. Palaparthy , M. S. Baghini , and M. J. C. Aslam , “Electrochemical Synthesis of Graphene Quantum Dots from Graphene Oxide at Room Temperature and Its Soil Moisture Sensing Properties,” Carbon 165 (2020): 9–17, 10.1016/j.carbon.2020.04.021.

[51] L. A. Ponomarenko , F. Schedin , M. I. Katsnelson , et al., “Chaotic Dirac Billiard in Graphene Quantum Dots,” Science 320 (2008): 356–358, 10.1126/science.1154663. 18420930

[52] Q. Gu , T. C. A. Ng , I. Zain , et al., “Chemical‐grafting of Graphene Oxide Quantum Dots (GOQDs) onto Ceramic Microfiltration Membranes for Enhanced Water Permeability and Anti‐organic Fouling Potential,” Applied Surface Science 502 (2020): 144128, 10.1016/j.apsusc.2019.144128.

[53] F. S. Carrillo , O. Z. Moran , F. Díaz Monge , and A. R. Juárez , “Luminescence Modification of Porous Silicon Decorated with GOQDs Synthesized by Green Chemistry,” AIMS Materials Science 12 (2025): 55–67, 10.3934/matersci.2025005.

[54] I. Chakraborti , U. Basak , S. Roy , M. Pakhira , A. Das , and D. P. J. N. Chatterjee , “Multi‐emissive Graphene Oxide Quantum Dots with Remarkable pH‐responsive Long‐wavelength Emission,” Nanoscale 17 (2025): 13387–13402, 10.1039/D5NR00526D. 40384314

[55] Z. Luo , G. Qi , K. Chen , et al., “Microwave‐Assisted Preparation of White Fluorescent Graphene Quantum Dots as a Novel Phosphor for Enhanced White‐Light‐Emitting Diodes,” Advanced Functional Materials 26 (2016): 2739–2744, 10.1002/adfm.201505044.

[56] S. Fan , S. Liu , Y. Xie , X. Zhou , and Y. Zhang , “Silk Fibroin/Graphene Quantum Dots Composite Memristor with Multi‐level Resistive Switching for Synaptic Emulators,” Journal of Materials Chemistry C 12 (2024): 3730–3738, 10.1039/D3TC04507B.

[57] L. Tang , R. Ji , X. Li , K. S. Teng , and S. P. Lau , “Size‐Dependent Structural and Optical Characteristics of Glucose‐Derived Graphene Quantum Dots,” Particle & Particle Systems Characterization 30 (2013): 523–531, 10.1002/ppsc.201200131.

[58] A. Allahbakhsh and A. R. Bahramian , “Self‐assembly of Graphene Quantum Dots into Hydrogels and Cryogels: Dynamic Light Scattering, UV–Vis Spectroscopy and Structural Investigations,” Journal of Molecular Liquids 265 (2018): 172–180, 10.1016/j.molliq.2018.05.123.

[59] J. Lu , P. S. E. Yeo , C. K. Gan , P. Wu , and K. P. Loh , “Transforming C60 Molecules into Graphene Quantum Dots,” Nature Nanotechnology 6 (2011): 247–252, 10.1038/nnano.2011.30. 21423185

[60] L. Shi , B. Wang , and S. Lu , “Efficient Bottom‐up Synthesis of Graphene Quantum Dots at an Atomically Precise Level,” Matter 6 (2023): 728–760, 10.1016/j.matt.2023.01.003.

[61] B. Lee , G. A. Stokes , A. Valimukhametova , et al., “Automated Approach to in Vitro Image‐Guided Photothermal Therapy with Top‐Down and Bottom‐Up‐Synthesized Graphene Quantum Dots,” Nanomaterials 13 (2023): 805, 10.3390/nano13050805. 36903683 PMC10005083

[62] K.‐C. Liu , P. Kumar Panda , B. Chandra Mallick , P.‐C. Yang , W.‐R. Liu , and C.‐T. Hsieh , “Solid‐phase Microwave Synthesis of High‐entropy Graphene Quantum Dots as Metal‐free Electrochemical Catalysts,” Applied Surface Science 648 (2024): 159061, 10.1016/j.apsusc.2023.159061.

[63] T. Vu Tuyet Nhung , H. Van Tuyen , N. Tran , and L. Xuan Hung , “S, N co‐doped Graphene Quantum Dots Fabricated by Rapid Microwave‐assisted Pyrolysis and Their Optical Properties,” Materials Today Communications 37 (2023): 107282, 10.1016/j.mtcomm.2023.107282.

[64] E. Haque , J. Kim , V. Malgras , et al., “Recent Advances in Graphene Quantum Dots: Synthesis, Properties, and Applications,” Small Methods 2 (2018): 1800050, 10.1002/smtd.201800050.

[65] A. Ghaffarkhah , E. Hosseini , M. Kamkar , et al., “Synthesis, Applications, and Prospects of Graphene Quantum Dots: a Comprehensive Review,” Small 18 (2022): 2102683, 10.1002/smll.202102683. 34549513

[66] X. Yan , X. Cui , and L.‐S. Li , “Synthesis of Large, Stable Colloidal Graphene Quantum Dots with Tunable Size,” Journal of the American Chemical Society 132 (2010): 5944–5945, 10.1021/ja1009376. 20377260

[67] Q. Li , S. Zhang , L. Dai , and L.‐S. Li , “Nitrogen‐Doped Colloidal Graphene Quantum Dots and Their Size‐Dependent Electrocatalytic Activity for the Oxygen Reduction Reaction,” Journal of the American Chemical Society 134 (2012): 18932–18935, 10.1021/ja309270h. 23126520

[68] H. Saleem , A. Saud , and S. J. Zaidi , “Sustainable Preparation of Graphene Quantum Dots from Leaves of Date Palm Tree,” ACS Omega 8 (2023): 28098–28108, 10.1021/acsomega.3c00694. 37576687 PMC10413365

[69] T. T. Hoang , H. P. Pham , and Q. T. Tran , “A Facile Microwave‐Assisted Hydrothermal Synthesis of Graphene Quantum Dots for Organic Solar Cell Efficiency Improvement,” Journal of Nanomaterials 2020 (2020): 1, 10.1155/2020/3207909.

[70] R. L. Calabro , D.‐S. Yang , and D. Y. Kim , “Controlled Nitrogen Doping of Graphene Quantum Dots through Laser Ablation in Aqueous Solutions for Photoluminescence and Electrocatalytic Applications,” ACS Applied Nano Materials 2 (2019): 6948–6959, 10.1021/acsanm.9b01433.

[71] S.‐Y. Kang , H. Yin , K.‐Q. Zhang , X. Chen , and K.‐Z. Wang , “Chemosensing Properties and Logic Gate Behaviors of Graphene Quantum Dot‐appended Terpyridine,” Materials Science and Engineering: C 99 (2019): 657–668, 10.1016/j.msec.2019.02.014. 30889739

[72] K. Li , M. Ji , R. Chen , Q. Jiang , J. Xia , and H. Li , “Construction of Nitrogen and Phosphorus co‐doped Graphene Quantum Dots/Bi_5_O_7_I Composites for Accelerated Charge Separation and Enhanced Photocatalytic Degradation Performance,” Chinese Journal of Catalysis 41 (2020): 1230–1239, 10.1016/S1872-2067(20)63531-8.

[73] R. Zhang , W. Shen , M. Zhong , J. Zhang , and S. Guo , “Carbon Nanofibers Cross‐Linked and Decorated with Graphene Quantum Dots as Binder‐Free Electrodes for Flexible Supercapacitors,” The Journal of Physical Chemistry C 125 (2021): 143–151, 10.1021/acs.jpcc.0c08803.

[74] Q. Ge , W.‐H. Kong , X.‐Q. Liu , et al., “Hydroxylated Graphene Quantum Dots as Fluorescent Probes for Sensitive Detection of Metal Ions,” International Journal of Minerals, Metallurgy and Materials 27 (2020): 91–99, 10.1007/s12613-019-1908-4.

[75] K. Rahimpour and R. Teimuri‐Mofrad , “Novel Hybrid Supercapacitor Based on Ferrocenyl Modified Graphene Quantum Dot and Polypyrrole Nanocomposite,” Electrochimica Acta 345 (2020): 136207, 10.1016/j.electacta.2020.136207.

[76] M. S. Islam , Y. Deng , L. Tong , et al., “In‐situ Direct Grafting of Graphene Quantum Dots Onto Carbon Fibre by Low Temperature Chemical Synthesis for High Performance Flexible Fabric Supercapacitor,” Materials Today Communications 10 (2017): 112–119, 10.1016/j.mtcomm.2016.11.002.

[77] Y. Wang , X. Liu , W. Kong , L. Wang , and Y. Li , “Post‐epoxidation of Graphene Quantum Dots,” Chemical Physics Letters 706 (2018): 140–144, 10.1016/j.cplett.2018.05.082.

[78] S. V. Giofrè , M. Tiecco , C. Celesti , et al., “Eco‐Friendly 1,3‐Dipolar Cycloaddition Reactions on Graphene Quantum Dots in Natural Deep Eutectic Solvent,” Nanomaterials (Basel, Switzerland) 10 (2020), 10.3390/nano10122549. PMC7765906 33352966

[79] X. Xu , G. He , S. Jiang , et al., “High Performance Enhancement‐mode Thin‐film Transistor with Graphene Quantum Dot‐decorated in_2_O_3_ Channel Layers,” RSC Advances 12 (2022): 14986–14997, 10.1039/D2RA01051H. 35702432 PMC9115870

[80] S. Gu , C.‐T. Hsieh , Y.‐Y. Tsai , et al., “Sulfur and Nitrogen Co‐Doped Graphene Quantum Dots as a Fluorescent Quenching Probe for Highly Sensitive Detection toward Mercury Ions,” ACS Applied Nano Materials 2 (2019): 790–798, 10.1021/acsanm.8b02010.

[81] J. Zhang , L. Chen , F. Zhu , et al., “Structure Evolution‐Driven Carrier Transport Engineering in Carbon Frameworks for High‐Performance all‐Carbon Photodetectors,” Advanced Functional Materials 36 (2026): 18271, 10.1002/adfm.202518271.

[82] K. Wu , Y. Wang , Y. Wan , et al., “Nitrogen and Oxygen Co‐Doped Graphene Quantum Dots as a Trace Amphipathic Additive for Dendrite‐Free Zn Anodes,” Advanced Functional Materials 35 (2025): 2412027, 10.1002/adfm.202412027.

[83] Y. Wang , T. Abdiryim , R. Jamal , F. Xu , Q. Zhang , and Y. Liu , “Achieving High‐performance Flexible UV Photodetectors: Modulating Oxygen Vacancies via Hydroxymethylated PEDOT and Surface Functionalities of N‐doped Graphene Quantum Dots,” Carbon 242 (2025): 120470, 10.1016/j.carbon.2025.120470.

[84] E. Zhang , T. Sun , B. Ge , et al., “High‐performance Solar‐blind Photodetector with Graphene and Nitrogen‐doped Reduced Graphene Oxide Quantum Dots (rGOQDs),” Materials Express 8 (2018): 105–111, 10.1166/mex.2018.1410.

[85] F. P. N. Inbanathan , K. L. A. Cimatu , D. C. Ingram , et al., “Paramagnetism in Microwave‐Synthesized Metal‐Free Nitrogen‐Doped Graphene Quantum Dots,” Materials 16 (2023): 3410, 10.3390/ma16093410. 37176291 PMC10179833

[86] G. Martins , A. L. S. Galvan , M. G. P. Valenga , T. A. Cardozo Martins , M. F. Bergamini , and L. H. Marcolino‐Junior , “Nitrogen‐Doped Graphene Quantum Dots (N‐GQDs): a Promising Material for the Development of Electrochemical Immunosensors,” ACS Applied Nano Materials 8 (2025): 5908–5918, 10.1021/acsanm.4c06568.

[87] D. Shen , T. Lan , D. Qiao , et al., “Tunable Photoluminescent Nitrogen‐Doped Graphene Quantum Dots at the Interface for High‐Efficiency Perovskite Solar Cells,” ACS Applied Nano Materials 7 (2024): 2232–2243, 10.1021/acsanm.3c05672.

[88] R. Das , S. Parveen , A. Bora , and P. K. Giri , “Origin of High Photoluminescence Yield and High SERS Sensitivity of Nitrogen‐doped Graphene Quantum Dots,” Carbon 160 (2020): 273–286, 10.1016/j.carbon.2020.01.030.

[89] H. Shah , W. Xie , Y. Wang , et al., “Preparation of Blue‐ and Green‐emissive Nitrogen‐doped Graphene Quantum Dots from Graphite and Their Application in Bioimaging,” Materials Science and Engineering: C 119 (2021): 111642, 10.1016/j.msec.2020.111642. 33321680

[90] S. Kang , Y. K. Jeong , K. H. Jung , et al., “One‐step Synthesis of Sulfur‐incorporated Graphene Quantum Dots Using Pulsed Laser Ablation for Enhancing Optical Properties,” Optics Express 28 (2020): 21659–21667, 10.1364/OE.398124. 32752439

[91] Y. Luo , M. Li , L. Sun , et al., “High Fluorescent Sulfur Regulating Graphene Quantum Dots with Tunable Photoluminescence Properties,” Journal of Colloid and Interface Science 529 (2018): 205–213, 10.1016/j.jcis.2018.06.016. 29894939

[92] Y. Li , S. Li , Y. Wang , et al., “Electrochemical Synthesis of Phosphorus‐doped Graphene Quantum Dots for Free Radical Scavenging,” Physical Chemistry Chemical Physics 19 (2017): 11631–11638, 10.1039/C6CP06377B. 28430285

[93] J. Qian , C. Shen , J. Yan , F. Xi , X. Dong , and J. Liu , “Tailoring the Electronic Properties of Graphene Quantum Dots by P Doping and Their Enhanced Performance in Metal‐Free Composite Photocatalyst,” The Journal of Physical Chemistry C 122 (2018): 349–358, 10.1021/acs.jpcc.7b08702.

[94] G. Wang , A. Xu , P. He , et al., “Green Preparation of Lattice Phosphorus Doped Graphene Quantum Dots with Tunable Emission Wavelength for Bio‐imaging,” Materials Letters 242 (2019): 156–159, 10.1016/j.matlet.2019.01.139.

[95] S. Kadian , G. Manik , N. Das , P. Nehra , R. P. Chauhan , and P. Roy , “Synthesis, Characterization and Investigation of Synergistic Antibacterial Activity and Cell Viability of Silver–sulfur Doped Graphene Quantum Dot (Ag@ S‐GQDs) Nanocomposites,” Journal of Materials Chemistry B 8 (2020): 3028–3037, 10.1039/C9TB02823D. 32186305

[96] T. Fan , G. Zhang , L. Jian , et al., “Facile Synthesis of Defect‐rich Nitrogen and Sulfur Co‐doped Graphene Quantum Dots as Metal‐free Electrocatalyst for the Oxygen Reduction Reaction,” Journal of Alloys and Compounds 792 (2019): 844–850, 10.1016/j.jallcom.2019.04.097.

[97] F. Shi , Q. Liu , J. Zhang , et al., “One‐Pot Synthesis of an FeS@GQDs Composite for Lithium Storage with Coal Tar Pitch as ″Natural GQDs″,” Energy & Fuels 36 (2022): 2130–2139, 10.1021/acs.energyfuels.1c03598.

[98] Z. Guo , H. Wu , M. Li , T. Tang , J. Wen , and X. Li , “Phosphorus‐doped Graphene Quantum Dots Loaded on TiO_2_ for Enhanced Photodegradation,” Applied Surface Science 526 (2020): 146724, 10.1016/j.apsusc.2020.146724.

[99] L. Goswami , N. Aggarwal , R. Verma , et al., “Graphene Quantum Dot‐Sensitized ZnO‐Nanorod/GaN‐Nanotower Heterostructure‐Based High‐Performance UV Photodetectors,” ACS Applied Materials & Interfaces 12 (2020): 47038–47047, 10.1021/acsami.0c14246. 32957784

[100] H. Chen , Q. Luo , T. Liu , et al., “Boosting Multiple Interfaces by Co‐Doped Graphene Quantum Dots for High Efficiency and Durability Perovskite Solar Cells,” ACS Applied Materials & Interfaces 12 (2020): 13941–13949, 10.1021/acsami.9b23255. 32079392

[101] S. Xiong , W. Wang , G. Fu , et al., “Graphene Quantum Dot Modified Bi_2_WO_6_ with Enhanced Photocatalytic Activity by Reinforcing the Charge Separation,” New Journal of Chemistry 44 (2020): 19220–19226, 10.1039/D0NJ03569F.

[102] X. Yu , Z. Zhao , N. Ren , et al., “Top or Bottom, Assembling Modules Determine the Photocatalytic Property of the Sheetlike Nanostructured Hybrid Photocatalyst Composed with Sn_3_O_4_ and rGO (GQD),” ACS Sustainable Chemistry & Engineering 6 (2018): 11775–11782, 10.1021/acssuschemeng.8b02030.

[103] X. Liu , Z. Zhou , T. Wang , C. Ma , and Y. Yan , “N‐doped Graphene Quantum Dots for Enhancing Multi‐level Bi_2_Ti_2_O_7_ Spheres Photocatalytic Activity via Electronic Trapping,” Journal of Dispersion Science and Technology 43 (2022): 639–648, 10.1080/01932691.2020.1844735.

[104] P. Ding , J. Di , X. Chen , et al., “S, N Codoped Graphene Quantum Dots Embedded in (BiO)_2_CO_3_: Incorporating Enzymatic‐Like Catalysis in Photocatalysis,” ACS Sustainable Chemistry & Engineering 6 (2018): 10229–10240, 10.1021/acssuschemeng.8b01552.

[105] D. S. Kuzmichev , A. G. Chernikova , M. G. Kozodaev , and A. M. Markeev , “Resistance Switching Peculiarities in Nonfilamentary Self‐Rectified TiN/Ta_2_O_5_/Ta and TiN/HfO_2_/Ta_2_O_5_/Ta Stacks,” Physica Status Solidi (A) Applications and Materials Science 217 (2020): 1900952, 10.1002/pssa.201900952.

[106] J. Yang , L. Hu , L. Shen , et al., “Optically Driven Intelligent Computing with ZnO Memristor,” Fundamental Research 4 (2024): 158–166, 10.1016/j.fmre.2022.06.019. 38933832 PMC11197590

[107] X. Wang , Z. Zhu , W. Li , et al., “Organic Memristor Based on Graphene Oxide Quantum Dots: PEDOT‐PSS Composite Film,” Materials Science in Semiconductor Processing 198 (2025): 109798, 10.1016/j.mssp.2025.109798.

[108] L. Kou , N. Ye , A. Waheed , et al., “High Sensitivity and Wide Response Range Artificial Synapse Based on Polyimide with Embedded Graphene Quantum Dots,” Scientific Reports 13 (2023): 8194, 10.1038/s41598-023-35183-8. 37210533 PMC10199894

[109] S. Yuan , B. Qiu , K. Amina , et al., “Robust and Low‐Power‐Consumption Black Phosphorus–Graphene Artificial Synaptic Devices,” ACS Applied Materials & Interfaces 14 (2022): 21242–21252, 10.1021/acsami.2c03667. 35499243

[110] Z. Zheng , X. Yang , L. Lv , et al., “Artificial Oxyanion Reservoir Accelerates Oriented Ionic Migration in MXene‐based Synaptic Memristor for Neuromorphic Computing,” Surfaces and Interfaces 63 (2025): 106315, 10.1016/j.surfin.2025.106315.

[111] A. Younis , D. Chu , X. Lin , J. Yi , F. Dang , and S. Li , “High‐Performance Nanocomposite Based Memristor with Controlled Quantum Dots as Charge Traps,” ACS Applied Materials & Interfaces 5 (2013): 2249–2254, 10.1021/am400168m. 23470212

[112] M. Li , M. Li , H. An , et al., “Highly Reliable Performance of Flexible Synaptic Devices Based on PVP–GO QD Nanocomposites due to the Formation of Directional Filaments,” ACS Applied Materials & Interfaces 16 (2024): 3621–3630, 10.1021/acsami.3c12615. 38197805

[113] X. Yan , L. Zhang , H. Chen , et al., “Graphene Oxide Quantum Dots Based Memristors with Progressive Conduction Tuning for Artificial Synaptic Learning,” Advanced Functional Materials 28 (2018): 1803728, 10.1002/adfm.201803728.

[114] A. S. Sokolov , M. Ali , R. Riaz , Y. Abbas , M. J. Ko , and C. Choi , “Silver‐Adapted Diffusive Memristor Based on Organic Nitrogen‐Doped Graphene Oxide Quantum Dots (N‐GOQDs) for Artificial Biosynapse Applications,” Advanced Functional Materials 29 (2019): 1807504, 10.1002/adfm.201807504.

[115] A. Nawaz , L. Merces , L. M. Ferro , P. Sonar , and C. C. B. Bufon , “Impact of Planar and Vertical Organic Field‐Effect Transistors on Flexible Electronics,” Advanced Materials 35 (2023): 2204804, 10.1002/adma.202204804. 36124375

[116] Y. Shi , Y. Ni , X. Zhao , et al., “Ultralow‐power Consumption Bimodal Synaptic Transistors for High‐efficiency Neuromorphic Vision System,” Journal of Colloid and Interface Science 702 (2026): 139012, 10.1016/j.jcis.2025.139012. 40972300

[117] J. Pei , L. Song , P. Liu , et al., “Scalable Synaptic Transistor Memory from Solution‐Processed Carbon Nanotubes for High‐Speed Neuromorphic Data Processing,” Advanced Materials 37 (2025): 2312783, 10.1002/adma.202312783. 39468862 PMC11733720

[118] Y. Yun , J. Park , M. Kong , et al., “Optoelectronic Neuromorphic System Based on Amorphous Indium–Gallium–Zinc–Oxide Thin‐Film Transistor for Spiking Neural Networks,” Advanced Intelligent Systems 8 (2025): 2500402, 10.1002/aisy.202500402.

[119] Y. Ji , J. Kim , A.‐N. Cha , et al., “Graphene Quantum Dots as a Highly Efficient Solution‐processed Charge Trapping Medium for Organic Nano‐floating Gate Memory,” Nanotechnology 27 (2016): 145204, 10.1088/0957-4484/27/14/145204. 26905768

[120] Y.‐H. Kim , E. Y. Lee , H. H. Lee , and T. S. Seo , “Characteristics of Reduced Graphene Oxide Quantum Dots for a Flexible Memory Thin Film Transistor,” ACS Applied Materials & Interfaces 9 (2017): 16375–16380, 10.1021/acsami.7b00714. 28445035

[121] D.‐C. Hu , R. Yang , L. Jiang , and X. Guo , “Memristive Synapses with Photoelectric Plasticity Realized in ZnO_1–x_/AlOy Heterojunction,” ACS Applied Materials & Interfaces 10 (2018): 6463–6470, 10.1021/acsami.8b01036. 29388420

[122] P. Zhang , C. Xu , G. Chen , et al., “Photodetection‐synaptic Transistor Based on 2D Perovskite Ferroelectrics for Color‐sensitive Camouflage Object Recognition,” Device 3 (2025): 100757, 10.1016/j.device.2025.100757.

[123] X. Zhu and W. D. Lu , “Optogenetics‐Inspired Tunable Synaptic Functions in Memristors,” ACS Nano 12 (2018): 1242–1249, 10.1021/acsnano.7b07317. 29357245

[124] Y. Wang , Z. Lv , J. Chen , et al., “Photonic Synapses Based on Inorganic Perovskite Quantum Dots for Neuromorphic Computing,” Advanced Materials 30 (2018): 1802883, 10.1002/adma.201802883. 30063261

[125] B. H. Jeong , J. Lee , S. W. Kim , and H. J. Park , “Flexible Photonic Synaptic Transistors with UV Responsivity via Graphene Quantum Dots for Neuromorphic Vision Systems,” ACS Applied Optical Materials 3 (2025): 1870–1880, 10.1021/acsaom.5c00234.

[126] Y. Kim , S. Cho , H. Kim , et al., “Graphene Quantum Dot (GQD)‐induced Photovoltaic and Photoelectric Memory Elements in a Pentacene/GQD Field Effect Transistor as a Probe of Functional Interface,” Journal of Physics D: Applied Physics 50 (2017): 365303, 10.1088/1361-6463/aa7e3f.

[127] M. Ali , A. Sokolov , M. J. Ko , and C. Choi , “Optically Excited Threshold Switching Synapse Characteristics on Nitrogen‐doped Graphene Oxide Quantum Dots (N‐GOQDs),” Journal of Alloys and Compounds 855 (2021): 157514, 10.1016/j.jallcom.2020.157514.

[128] B. Wang , J. Wang , and S. Lu , “Carbon Dots: Small Materials with Big Impacts on Optoelectronic Devices,” Aggregate 6 (2025): 70212, 10.1002/agt2.70212.

[129] M. Chaudhary , C. Xin , Z. Hu , et al., “Nitrogen‐Doped Carbon Quantum Dots on Graphene for Field‐Effect Transistor Optoelectronic Memories,” Advanced Electronic Materials 9 (2023): 2300159, 10.1002/aelm.202300159.

[130] L. Wang , Y. Zhang , P. Zhang , and D. Wen , “Physical Transient Photoresistive Variable Memory Based on Graphene Quantum Dots,” Nanomaterials 12 (2022): 3976, 10.3390/nano12223976. 36432261 PMC9695640

[131] X. Li , Y. Pei , Y. Zhao , et al., “Memristors Based on Carbon Dots for Learning Activities in Artificial Biosynapse Applications,” Materials Chemistry Frontiers 6 (2022): 1098–1106, 10.1039/D2QM00151A.

[132] S.‐Y. Li and L. He , “Recent Progresses of Quantum Confinement in Graphene Quantum Dots,” Frontiers of Physics 17 (2021): 33201, 10.1007/s11467-021-1125-2.

[133] P. Vu Nhat , N. V. Duy , T. N. Tran , et al., “Optoelectronic Properties of Nitrogen‐Doped Hexagonal Graphene Quantum Dots: a First‐Principles Study,” ACS Omega 9 (2024): 20056–20065, 10.1021/acsomega.3c10501. 38737018 PMC11079900

[134] F. Zhao , T. Zhang , S. Sui , and Z. Chen , “Influence on the Optical and Electronic Properties of Graphene Quantum Dots Originating from the S‐Doping Site: a Theoretical Investigation,” The Journal of Physical Chemistry A 129 (2025): 4357–4363, 10.1021/acs.jpca.5c00370. 40347170

[135] X. Li , S. P. Lau , L. Tang , R. Ji , and P. Yang , “Sulphur Doping: a Facile Approach to Tune the Electronic Structure and Optical Properties of Graphene Quantum Dots,” Nanoscale 6 (2014): 5323–5328, 10.1039/C4NR00693C. 24699893

[136] N. Eslami , S. H. H. Nemati , and M. H. Moaiyeri , “In‐memory Computing Platform Based on a Novel Single‐MTJ Non‐volatile SRAM Design for Intermittent AI Computation,” Computers and Electrical Engineering 128 (2025): 110755, 10.1016/j.compeleceng.2025.110755.

[137] G. Poply , Manisha , N. Verma , et al., “Enhancement of the Performance of Non‐volatile Resistive Switching Memory and High Dielectric Constant through Chemical Synthesis Techniques of Multi‐walled Carbon Nanotube (MWCNT)‐TiO_2_/Polymer Nanocomposites,” Inorganic Chemistry Communications 182 (2025): 115655, 10.1016/j.inoche.2025.115655.

[138] M. Jangid , K. Kaushik , G. M. Khanal , et al., “Design and Analysis of Multi‐layer ZrO_2_‐based Resistive Random Access Memory (RRAM) for next‐generation Non‐volatile Memory,” Micro and Nanostructures 208 (2025): 208377, 10.1016/j.micrna.2025.208377.

[139] M. Farronato , P. Mannocci , M. Melegari , S. Ricci , C. M. Compagnoni , and D. Ielmini , “Reservoir Computing with Charge‐Trap Memory Based on a MoS_2_ Channel for Neuromorphic Engineering,” Advanced Materials 35 (2023): 2205381, 10.1002/adma.202205381. 36222391

[140] C. Liu , J. Pan , Q. Yuan , et al., “Highly Reliable Van Der Waals Memory Boosted by a Single 2D Charge Trap Medium,” Advanced Materials 36 (2024): 2305580, 10.1002/adma.202305580. 37882079

[141] T. Yang , H. Wang , B. Zhang , and X. Yan , “Enhanced Memory Characteristics of Charge Trapping Memory by Employing Graphene Oxide Quantum Dots,” Applied Physics Letters 116 (2020): 103501, 10.1063/1.5135623.

[142] L. Li , “Graphene Oxide: Graphene Quantum Dot Nanocomposite for Better Memristic Switching Behaviors,” Nanomaterials 10 (2020): 1448, 10.3390/nano10081448. 32722171 PMC7466482

[143] U. I. Bature , H. Abbas , A. Alzahrani , A. Nisar , F. Bashir , and F. Zahoor , “Analysis of Resistive Switching Properties in TiO2‐based RRAM Device for Neuromorphic Computing Applications,” Applied Materials Today 47 (2025): 102916, 10.1016/j.apmt.2025.102916.

[144] Z. Wang , Y. Song , G. Zhang , et al., “Advances of Embedded Resistive Random Access Memory in Industrial Manufacturing and Its Potential Applications,” International Journal of Extreme Manufacturing 6 (2024): 032006, 10.1088/2631-7990/ad2fea.

[145] T.‐J. Wang , C.‐Y. Li , P.‐A. Shih , et al., “Investigations of SiC Doping Effects on the Performance Improvement of ZnO‐based RRAMs at Extremely Temperature,” Materials Science in Semiconductor Processing 197 (2025): 109717, 10.1016/j.mssp.2025.109717.

[146] A. I. Ivanov , N. A. Nebogatikova , I. A. Kotin , S. A. Smagulova , and I. V. Antonova , “Resistive Switching Effects in Fluorinated Graphene Films with Graphene Quantum Dots Enhanced by Polyvinyl Alcohol,” Nanotechnology 30 (2019): 255701, 10.1088/1361-6528/ab0cb3. 30836347

[147] S. Ali , J. Bae , C. H. Lee , K. H. Choi , and Y. H. Doh , “All‐printed and Highly Stable Organic Resistive Switching Device Based on Graphene Quantum Dots and Polyvinylpyrrolidone Composite,” Organic Electronics 25 (2015): 225–231, 10.1016/j.orgel.2015.06.040.

[148] X. Yan , L. Zhang , Y. Yang , et al., “Highly Improved Performance in Zr0.5Hf0.5O2 Films Inserted with Graphene Oxide Quantum Dots Layer for Resistive Switching Non‐volatile Memory,” Journal of Materials Chemistry C 5 (2017): 11046–11052, 10.1039/C7TC03037A.

[149] N. A. Nebogatikova , I. V. Antonova , A. I. Ivanov , et al., “Fluorinated Graphene Nanoparticles with 1–3 Nm Electrically Active Graphene Quantum Dots,” Nanotechnology 31 (2020): 295602, 10.1088/1361-6528/ab83b8. 32213679

[150] A. I. Ivanov , I. V. Antonova , N. A. Nebogatikova , and A. Olejniczak , “Memristive FG–PVA Structures Fabricated with the Use of High Energy Xe Ion Irradiation,” Materials 15 (2022): 2085, 10.3390/ma15062085. 35329539 PMC8950800

[151] A. Pisal Deshmukh , K. Patil , K. Barve , and T. Bhave , “Transient N‐GQDs/PVA Nanocomposite Thin Film for Memristor Application,” Nanotechnology 35 (2024): 265706, 10.1088/1361-6528/ad364b. 38513286

[152] S. Cruces , M. D. Ganeriwala , J. Lee , et al., “Volatile MoS_2_ Memristors with Lateral Silver Ion Migration for Artificial Neuron Applications,” Small Science 5 (2025): 2400523, 10.1002/smsc.202400523. 40395355 PMC12087773

[153] Y. Pei , B. Yang , X. Zhang , et al., “Ultra Robust Negative Differential Resistance Memristor for Hardware Neuron Circuit Implementation,” Nature Communications 16 (2025): 48, 10.1038/s41467-024-55293-9. PMC11696043 39746986

[154] Z. Wang , S. Joshi , S. Savel'ev , et al., “Fully Memristive Neural Networks for Pattern Classification with Unsupervised Learning,” Nature Electronics 1 (2018): 137–145, 10.1038/s41928-018-0023-2.

[155] X. Li , Y. Zhong , H. Chen , et al., “A Memristors‐Based Dendritic Neuron for High‐Efficiency Spatial‐Temporal Information Processing,” Advanced Materials 35 (2023): 2203684, 10.1002/adma.202203684. 35735048

[156] Y. Chen , G. Zhang , F. Liu , et al., “Revolutionizing Neuromorphic Computing with Memristor‐based Artificial Neurons,” Journal of Semiconductors 46 (2025): 061301, 10.1088/1674-4926/24110006.

[157] M. Kaźmierczak , S. Giannini , and S. Osella , “Photoinduced Energy and Electron Transfer at Graphene Quantum Dot/Azobenzene Interfaces,” Journal of Materials Chemistry C 12 (2024): 143–153, 10.1039/D3TC03667G.

[158] L.‐F. Liu , Z.‐R. Zhao , Q.‐J. Sun , G. Tang , X.‐G. Tang , and Y. Zhou , “A Bio‐inspired Tactile Sensor for Artificial Tactile Synapses,” Materials Today Physics 59 (2025): 101890, 10.1016/j.mtphys.2025.101890.

[159] Z. Xia , Y. Chen , L. Su , and H. Chen , “Bioinspired Artificial Optoelectronic Synapse for Encrypted Communication Realized via a MoSe_2_ Based MIS Structural Photodiode,” Journal of Materials Science & Technology 243 (2026): 129–138, 10.1016/j.jmst.2025.05.007.

[160] J. Li , H. Xu , S.‐Y. Sun , et al., “Enhanced Spiking Neural Network with Forgetting Phenomenon Based on Electronic Synaptic Devices,” Neurocomputing 408 (2020): 21–30, 10.1016/j.neucom.2019.09.030.

[161] C. Wang , W. He , Y. Tong , et al., “Memristive Devices with Highly Repeatable Analog States Boosted by Graphene Quantum Dots,” Small 13 (2017): 1603435, 10.1002/smll.201603435. 28296020

[162] C. Gao , H. Wang , Z. Zhu , et al., “A High‐Performance Memristor Device and Its Filter Circuit Application,” Physica Status Solidi (RRL)—Rapid Research Letters 14 (2020): 2000389, 10.1002/pssr.202000389.

[163] F. Duan , F. Fan , T. Huang , W. Li , S. Sun , and B. Zhang , “Graphene Quantum Dots Covalently Functionalized with Zinc Porphyrin for Digital‐analog Dual‐mode Memristors,” Nanoscale 17 (2025): 23363–23372, 10.1039/D5NR02683K. 41001792

[164] S. Y. Ryu , H. S. Kim , J. S. An , et al., “Electrical Synaptic Devices with a High Recognition Rate Based on Eco‐friendly Nanocomposites of a Poly(methyl methacrylate) Matrix Embedded with Graphene Quantum Dots for Neuromorphic Computing,” Organic Electronics 126 (2024): 106997, 10.1016/j.orgel.2024.106997.

[165] T. Tadros , G. P. Krishnan , R. Ramyaa , and M. Bazhenov , “Sleep‐Like Unsupervised Replay Reduces Catastrophic Forgetting in Artificial Neural Networks,” Nature Communications 13 (2022): 7742, 10.1038/s41467-022-34938-7. PMC9755223 36522325

[166] X. Zhang , C. Zhong , J. Zhang , T. Wang , and W. W. Y. Ng , “Robust Recurrent Neural Networks for Time Series Forecasting,” Neurocomputing 526 (2023): 143–157, 10.1016/j.neucom.2023.01.037.

[167] G. Dellaferrera , S. Woźniak , G. Indiveri , A. Pantazi , and E. Eleftheriou , “Introducing Principles of Synaptic Integration in the Optimization of Deep Neural Networks,” Nature Communications 13 (2022): 1885, 10.1038/s41467-022-29491-2. PMC8989917 35393422

[168] X. Fan , A. Chen , Z. Li , et al., “Metaplasticity‐Enabled Graphene Quantum Dot Devices for Mitigating Catastrophic Forgetting in Artificial Neural Networks,” Advanced Materials 37 (2025): 2411237, 10.1002/adma.202411237. 39648507

[169] G. Wang , R. Wang , W. Kong , and J. Zhang , “Simulation of Retinal Ganglion Cell Response Using Fast Independent Component Analysis,” Cognitive Neurodynamics 12 (2018): 615–624, 10.1007/s11571-018-9490-4. 30483369 PMC6233330

[170] L. Gu , S. Poddar , Y. Lin , et al., “A Biomimetic Eye with a Hemispherical Perovskite Nanowire Array Retina,” Nature 581 (2020): 278–282, 10.1038/s41586-020-2285-x. 32433619

[171] H.‐L. Park , H. Kim , D. Lim , et al., “Retina‐Inspired Carbon Nitride‐Based Photonic Synapses for Selective Detection of UV Light,” Advanced Materials 32 (2020): 1906899, 10.1002/adma.201906899. 31984573

[172] W. Zeng , M. Hou , H. Li , et al., “2D Covalent Organic Framework‐Based Heterostructure for Neuromorphic Visual Processing,” Advanced Functional Materials 35 (2025): 11687, 10.1002/adfm.202511687.

[173] X. Zhao , Y. Wu , Z. Xia , S. Chang , Y. Shang , and A. Cao , “A GQD‐based Composite Film as Photon Down‐converter in CNT/Si Solar Cells,” Nano Research 14 (2021): 3893–3899, 10.1007/s12274-021-3311-5.

[174] L. Wang , J. Yang , Y. Zhang , and D. Wen , “Dual‐Tunable Memristor Based on Carbon Nanotubes and Graphene Quantum Dots,” Nanomaterials 11 (2021): 2043, 10.3390/nano11082043. 34443874 PMC8401814

[175] L. Wang , S. Wei , J. Xie , Y. Ju , T. Yang , and D. Wen , “Artificial Synapses Based on an Optical/Electrical Biomemristor,” Nanomaterials 13 (2023): 3012, 10.3390/nano13233012. 38063708 PMC10708255

[176] T. Xie , Q. Wang , M. Li , et al., “Carbon Nanotube Optoelectronic Synapse Transistor Arrays with Ultra‐Low Power Consumption for Stretchable Neuromorphic Vision Systems,” Advanced Functional Materials 33 (2023): 2303970, 10.1002/adfm.202303970.

[177] S. Wang , J. Xu , W. Wang , et al., “Skin Electronics from Scalable Fabrication of an Intrinsically Stretchable Transistor Array,” Nature 555 (2018): 83–88, 10.1038/nature25494. 29466334

[178] B. Huang , J. Pan , L. Lan , et al., “Emotion‐modulated Visual Perception in Ultra‐low‐energy Flexible Neuromorphic Synaptic Transistors Enabled by Directionally Polarized Lactam‐based Polymer Electrets,” Nano Energy 142 (2025): 111202, 10.1016/j.nanoen.2025.111202.

[179] L. Gao , M. Wu , X. Yu , and J. Yu , “Device Design Principles and Bioelectronic Applications for Flexible Organic Electrochemical Transistors,” International Journal of Extreme Manufacturing 6 (2024): 012005, 10.1088/2631-7990/acfd69.

[180] J. Kim , S. Lee , J. Yoon , and D. Son , “Soft Sensory‐neuromorphic System for Closed‐loop Neuroprostheses,” International Journal of Extreme Manufacturing 7 (2025): 042001, 10.1088/2631-7990/adb9aa.

[181] S. Ali , A. Hassan , G. Hassan , J. Bae , and C. H. Lee , “Flexible Frequency Selective Passive Circuits Based on Memristor and Capacitor,” Organic Electronics 51 (2017): 119–127, 10.1016/j.orgel.2017.09.012.

[182] L. Wang , Y. Wang , J. Yang , W. Li , and D. Wen , “Bioresistive Random Access Memory with an in‐memory Computing Function Based on Graphene Quantum Dots,” New Journal of Chemistry 47 (2023): 9459–9463, 10.1039/D3NJ00076A.

[183] S. Sung , J. H. Park , C. Wu , and T. W. Kim , “Biosynaptic Devices Based on Chicken Egg Albumen:Graphene Quantum Dot Nanocomposites,” Scientific Reports 10 (2020): 1255, 10.1038/s41598-020-57966-z. 31988397 PMC6985135

[184] P. Roy , A. P. Periasamy , C. Chuang , et al., “Plant Leaf‐derived Graphene Quantum Dots and Applications for White LEDs,” New Journal of Chemistry 38 (2014): 4946–4951, 10.1039/C4NJ01185F.

[185] N.‐J. Kuo , Y.‐S. Chen , C.‐W. Wu , C.‐Y. Huang , Y.‐H. Chan , and I. W. P. Chen , “One‐Pot Synthesis of Hydrophilic and Hydrophobic N‐Doped Graphene Quantum Dots via Exfoliating and Disintegrating Graphite Flakes,” Scientific Reports 6 (2016): 30426, 10.1038/srep30426. 27452118 PMC4958986

[186] L. Wang , J. Yang , X. Zhang , and D. Wen , “High‐Performance Biomemristor Embedded with Graphene Quantum Dots,” Nanomaterials 13 (2023): 3021, 10.3390/nano13233021. 38063717 PMC10708532

[187] T. Chen , S. Yang , J. Wang , et al., “Flexible Artificial Memristive Synapse Constructed from Solution‐Processed MgO–Graphene Oxide Quantum Dot Hybrid Films,” Advanced Electronic Materials 7 (2021): 2000882, 10.1002/aelm.202000882.

[188] S. Ren , Z. Li , X. Liu , Y. Li , G. Cao , and J. Zhao , “Oxygen Migration Induced Effective Magnetic and Resistive Switching Boosted by Graphene Quantum Dots,” Journal of Alloys and Compounds 863 (2021): 158339, 10.1016/j.jallcom.2020.158339.

[189] F. Fan , B. Zhang , Y. Cao , X. Yang , J. Gu , and Y. Chen , “Conjugated Polymer Covalently Modified Graphene Oxide Quantum Dots for Ternary Electronic Memory Devices,” Nanoscale 9 (2017): 10610–10618, 10.1039/c7nr02809a. 28726942

[190] G. Zhou , B. Sun , X. Hu , et al., “Negative Photoconductance Effect: an Extension Function of the TiO_x_‐Based Memristor,” Advanced Science 8 (2021): 2003765, 10.1002/advs.202003765.

[191] Z. Xu , F. Li , C. Wu , et al., “Ultrathin Electronic Synapse Having High Temporal/Spatial Uniformity and an Al_2_O_3_/Graphene Quantum Dots/Al_2_O_3_ Sandwich Structure for Neuromorphic Computing,” NPG Asia Materials 11 (2019): 18, 10.1038/s41427-019-0118-x.

[192] X. Yan , H. Li , L. Zhang , et al., “Density Effects of Graphene Oxide Quantum Dots on Characteristics of Zr_0.5_Hf_0.5_O_2_ Film Memristors,” Applied Physics Letters 114 (2019), 10.1063/1.5089532.

